# Shape-Defined microPlates for the Sustained Intra-articular Release of Dexamethasone in the Management of Overload-Induced Osteoarthritis

**DOI:** 10.1021/acsami.1c02082

**Published:** 2021-07-01

**Authors:** Martina Di Francesco, Sean K. Bedingfield, Valentina Di Francesco, Juan M. Colazo, Fang Yu, Luca Ceseracciu, Elena Bellotti, Daniele Di Mascolo, Miguel Ferreira, Lauren E. Himmel, Craig Duvall, Paolo Decuzzi

**Affiliations:** † Laboratory of Nanotechnology for Precision Medicine, Istituto Italiano di Tecnologia, Via Morego 30, Genoa 16163, Italy; ‡ Department of Biomedical Engineering, Vanderbilt University, Nashville, Tennessee 37212, United States; § Department of Informatics, Bioengineering, Robotics and System Engineering, University of Genoa, Via Opera Pia 13, Genoa 16145, Italy; ∥ Materials Characterization Facility, Istituto Italiano di Tecnologia, Via Morego 30, Genova 16163, Italy; ⊥ Department of Pathology, Microbiology and Immunology, Vanderbilt University Medical Center, Nashville, Tennessee 37212, United States

**Keywords:** osteoarthritis, polymeric microparticles, mechanical properties, sustained release, drug depot

## Abstract

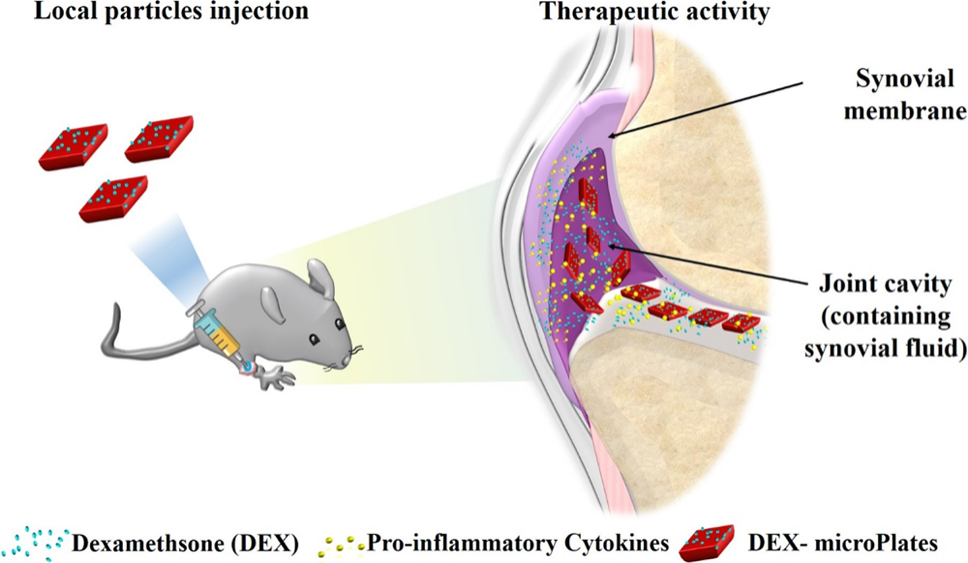

Osteoarthritis (OA) is treated with the intra-articular injection of steroids such as dexamethasone (DEX) to provide short-term pain management. However, DEX treatment suffers from rapid joint clearance. Here, 20 × 10 μm, shape-defined poly(d,l-lactide-co-glycolide)acid microPlates (μPLs) are created and intra-articularly deposited for the sustained release of DEX. Under confined conditions, DEX release is projected to persist for several months, with only ∼20% released in the first month. In a highly rigorous murine knee overload injury model (post-traumatic osteoarthritis), a single intra-articular injection of Cy5-μPLs is detected in the cartilage surface, infrapatellar fat pad/synovium, joint capsule, and posterior joint space up to 30 days. One intra-articular injection of DEX-μPL (1 mg kg^–1^) decreased the expression of interleukin (IL)-1β, tumor necrosis factor (TNF)-α, IL-6, and matrix metalloproteinase (MMP)-13 by approximately half compared to free DEX at 4 weeks post-treatment. DEX-μPL also reduced load-induced histological changes in the articular cartilage and synovial tissues relative to saline or free DEX. In sum, the μPLs provide sustained drug release along with the capability to precisely control particle geometry and mechanical properties, yielding long-lasting benefits in overload-induced OA. This work motivates further study and development of particles that provide combined pharmacological and mechanical benefits.

## 1 Introduction

Osteoarthritis (OA) is a prevalent disease that causes chronic pain and disability, especially among the elderly.^1,2^ OA can also affect younger patients, often due to post-traumatic osteoarthritis (PTOA), a form of OA which can occur after joint, ligament, and bone injury or surgery.^3^ PTOA, in opposition to idiopathic age-associated OA, has an identifiable triggering event that makes therapeutics for prevention clinically feasible. OA-associated mechanical wear or traumatic joint injury promotes increased expression of proinflammatory cytokines [e.g., interleukin (IL)-1β, IL-6, and tumor necrosis factor (TNF)-α] and matrix metalloproteinases (MMPs) in the affected joint. Excessive inflammation reduces the synthesis of extracellular matrix components and increases matrix degradation, driven principally by aggrecanases and MMPs, resulting in lesions on the articular cartilage surface that progress toward full cartilage erosion and complete joint dysfunction.^4,5^

Nonsteroidal anti-inflammatory drugs (NSAIDs) are typically the first line of pharmaceutical treatment but are only marginally effective at pain relief, can cause gastrointestinal complications, and do not slow cartilage deterioration.^6,7^ Also, one challenge for NSAID systemic administration is the insufficient accumulation in the joints, which are relatively avascular, making local injection a good alternative for increasing bioavailability and decreasing systemic exposure.^7^ In the current clinical practice, the Osteoarthritis Research Society International (OARSI) and the American College of Rheumatology recommend articular injection of anti-inflammatory corticosteroids for symptomatic knee OA with dexamethasone (DEX) as one of the five FDA-approved corticosteroids for this use.^7−9^ A limitation of the intra-articular injection of steroids is the lack of local retention as small molecules are cleared through the synovial vasculature and macromolecules drained through the lymphatics, leading to joint half-lives ranging from 1 to 4 h.^10^

Biomaterial depots offer a reliable approach for improving drug pharmacokinetics, particularly for treating chronic diseases.^11,12^ To this end, Flexion Therapeutics developed and recently achieved FDA approval for the sustained release of triamcinolone acetonide from poly(d,l-lactide-co-glycolide) acid (PLGA) microparticles.^13^ Note that this anti-inflammatory corticosteroid belongs to the same drug class as DEX. Biomaterials for mechanical cushioning in the knee are also applied clinically, with intra-articular injection of hyaluronic acid (HA) being the primary approach used to enhance the mechanical properties of the synovial fluid. HA injections are posited to reduce pain by increasing hydration, lubrication, and resistance to shear in the joint.^14,15^ However, even for larger-molecular-weight bio-macromolecules such as HA, the relief is short-lived as the half-life within the joint is approximately 1 day,^10^ and there is no strong evidence that HA provides palliative benefit over steroid injection.^7^ A promising preclinical approach to repair the cartilage structure and mechanical integrity, while also providing a sustained release depot, involves press-fitting drug-loaded, macroscopic gels into cartilage defects.^16^ This approach enables long-term (several months) release of DEX directly within the cartilaginous tissue and could be promising for late stage disease, where more invasive surgical intervention is justified.

Our group recently reported an injectable drug depot comprising shape-defined PLGA-based microplates (μPLs).^17^ These microconstructs exhibit distinct physicochemical properties, dictated by their size, shape, surface, and mechanical properties which can be simultaneously and independently tailored during the synthesis process. Size and shape control provides desirable formulation homogeneity and reproducibility, while the ability to tune particle surface and mechanical properties can provide application-specific benefits. Here, this technology is applied to produce DEX-loaded polymeric μPLs (DEX-μPLs) in order to increase drug exposure within the articular joint. After briefly describing the fabrication process and characterizing the morphological, mechanical, and pharmacological properties of DEX-μPLs, these particles are tested *in vitro* for their anti-inflammatory potential and *in vivo* for pharmacokinetics and their ability to reduce joint structural changes due to overload injury-associated PTOA.

## 2 Results

### 2.1 Synthesis and Characterization of Dexamethasone-loaded MicroPlates (DEX-μPLs)

**Figure 1:**
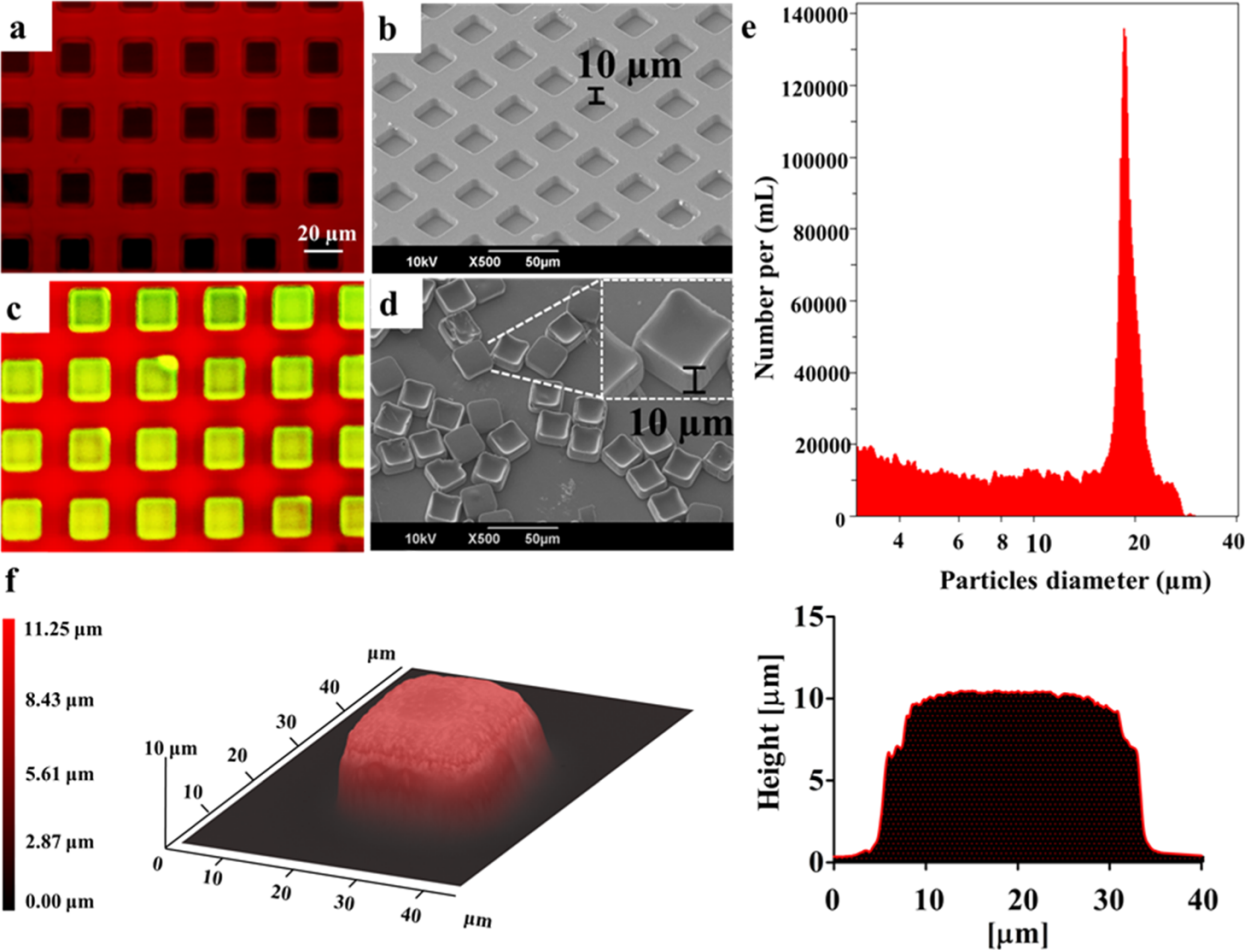
Geometrical characterization of μPLs. (a) Confocal microscopy and (b) SEM image of an empty PVA template; (c) confocal microscopy image of a PVA template (red) filled with a PLGA/CURC paste forming CURC-μPLs (green/yellow); (d) SEM image of individual μPLs released from the PVA template. The lateral inset shows a magnified and tilted view of the μPLs; (e) size distribution profile for μPLs *via* a Multisizer Coulter Counter analysis; (f) optical-profilometer topographic image of a μPL, where the red-level false coloring correlates with the local particle thickness. The plot on the right represents the cross-sectional profile of the μPL.

μPLs were generated using a sacrificial-template fabrication strategy.^17^ A silicon master template comprising a two-dimensional (2D) matrix of wells used to define the particles’ geometry was first built using a direct laser writing (DLW) method. Then, a polydimethylsiloxane (PDMS) template was generated by replicating the original master template. This intermediate PDMS template presents a 2D matrix of pillars with the same geometry as the particles. Finally, a poly(vinyl alcohol) (PVA) template was produced by replicating the PDMS template (Figure 1a,b). This sacrificial template presents a 2D matrix of wells, identical to that of the original master template, which are filled with the polymeric paste (PLGA) containing the drug payload (Figure 1c). Then, the μPLs were released in an aqueous solution by progressively dissolving the sacrificial PVA template (Figure 1d). The microscopy images presented in Figure 1d demonstrate the defined and uniform geometry of the μPLs, presenting a length and width of 20 μm and a height of 10 μm.

Particles loaded with curcumin (CURC) (CURC-μPLs) for visualization purposes (yellow/green) appeared to have a well-defined geometry clearly defined by the wells in the PVA template, which appears red due to the dispersion of a rhodamine B fluorescent probe (Figure 1c). Dissolution of the PVA template in water resulted in the release of the μPLs, which were then characterized by electron microscopy and a multisizer particle counter (Figure 1d,e). A scanning electron microscopy (SEM) image of the particles from a 30° tilted view (Figure 1d) shows that the size and cuboidal shape of the particles match that of the original template. The analysis of the μPL number and size distribution using a Multisizer (Figure 1e) showed a single peak around ∼20 μm with a relatively narrow distribution. A topographical analysis, under hydrated conditions, with an optical profilometer (Figure 1f) confirmed the values of the particle thickness and the overall geometry. This can be appreciated *via* the false-coloring 3D reconstruction and the cross-sectional profile, both shown in Figure 1f. Similar profilometric data were also generated for lyophilized μPLs (Figure S1) demonstrating an ∼10% variation (see Supporting Information and Table S2) in volume between the two configurations (fully hydrated particles *vs* lyophilized particles).

### 2.2 Pharmacological and Mechanical Characterization of Dexamethasone-loaded MicroPlates (DEX-μPLs)

**Figure 2:**
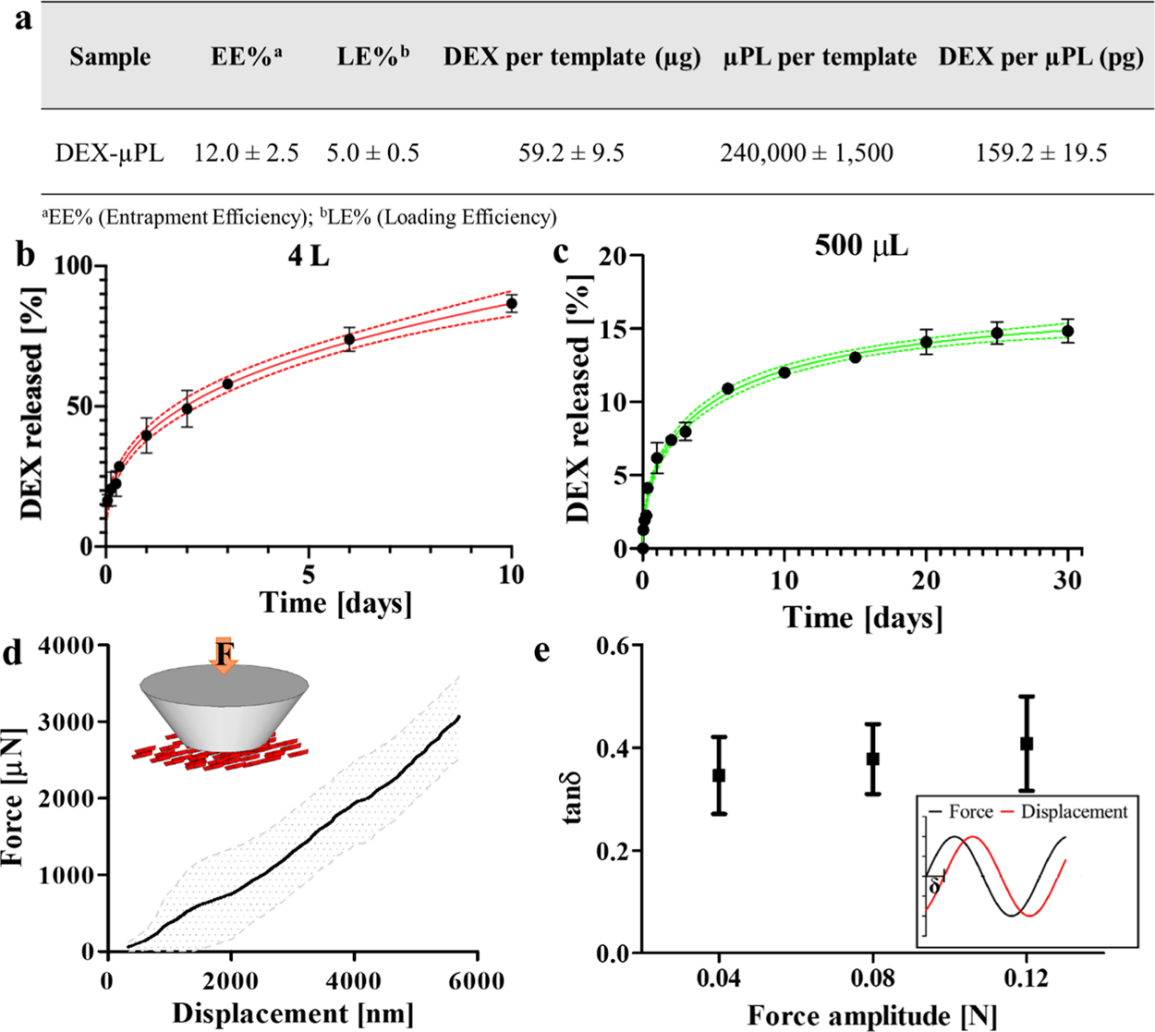
Drug loading, release kinetics, and mechanical characterization of DEX-loaded microPlates (DEX-μPLs). (a) DEX-μPL fabrication yield and drug loading characterization; (b) DEX release profile from μPLs under the sink condition (4 L, red line) and Weibull function fitting (red line; 95% confidence band); (c) DEX release profile from μPLs under confined conditions (500 μL; green line; 95% confident band) and Weibull function fitting; (d) force-displacement curve for a flat punch indentation experiment on μPLs (average curve and standard deviation). In the inset, a schematic of the experimental setup is provided; (e) mechanical damping of μPLs upon cyclic loading (frequency 5 Hz) as a function of the force oscillation amplitude. In the inset, a schematic of the testing routine highlights the phase angle δ—dissipation parameter. Results are presented as the average ± standard deviation (SD) (*n* = 3).

The DEX-μPL fabrication process was characterized in terms of fabrication yield (number of particles per template), entrapment efficiency (EE), loading efficiency (LE), and amount of DEX per μPL (Figure 2a). As previously reported,^17^ the fabrication yield, calculated as the ratio between the number of μPLs collected after PVA dissolution and the theoretical number of wells in a template, was approximately 40%. The amount of DEX loaded per template was 59.2 ± 9.5 μg, with LE and EE of 5.0 ± 0.5 and 12.0 ± 2.5%, respectively. These measured EE and LE values are in line with those in other published literature studies on PLGA microparticle drug encapsulation.^18,19^ The amount of DEX loaded per particle was 159.2 ± 19.5 pg. Considering the theoretical volume of a single μPL being 4 × 10^–3^ nL (i.e., volume = *L* × *W* × *H* = 20 μm × 20 μm × 10 μm = 4000 μm^3^ = 4 × 10^–3^ nL), the DEX volumetric concentration per μPL was approximately 40 kg m^–3^. *In vitro* drug release kinetics were measured under two different conditions, namely, 4 L, which approximates an infinite sink, and 500 μL, which approximates confinement within the synovial cavity after intra-articular injection.^20^ Briefly, DEX-μPLs were kept in two different volumes of phosphate-buffered saline (PBS) buffer (1×, pH 7.4), 4 L and 500 μL, at 37 ± 2 °C under magnetic stirring. For the 4 L condition, three samples at each time point were collected and centrifuged down, and pellets were isolated and dissolved in acetonitrile/H_2_O (1:1, v/v) to release the remaining DEX. The resulting solution was analyzed *via* high-performance liquid chromatography (HPLC) to quantify the drug loaded in the μPLs at each time point. For the 500 μL condition, three samples at each time point (Figure 2c) were collected, centrifuged down, and resuspended with 500 μL of fresh PBS buffer. Then, the supernatants were collected, mixed with acetonitrile, and analyzed *via* HPLC for drug content. Both release conditions showed profiles consistent with diffusion-controlled release but with different percentages of released DEX under the two different conditions (Figure 2b,c). Specifically, there was a 30% burst release of DEX within the first 8 h under the sink condition. Conversely, in the more physiologically relevant condition of 500 μL, only ∼4% of DEX was released within the first 8 h. Under both conditions, the remaining encapsulated DEX was released at a relatively constant rate, yielding approximately 85% release after 10 days under the sink condition and approximately 20% after 30 days in 500 μL. This sustained drug release from the μPLs is expected to provide a significant extension in the DEX dwell time within the joint cavity as opposed to its free formulation, which typically has a half-life of 1–4 h.^10^

The initial, faster phase of release is likely associated to DEX molecules residing in the vicinity of the particle surface that would diffuse out more rapidly. On the other hand, the second, slower release phase should be ascribed to the sustained diffusion of the DEX molecules from the particle interior and the progressive degradation of the PLGA matrix of the μPLs.^21,22^ To further analyze the mechanism of release, the profiles of Figure 2b,c were fitted with the Weibull function to derive the corresponding coefficients a and b. Specifically, values for b smaller than 0.75 would suggest a release kinetic dominated by Fickian diffusion rather than matrix degradation or swelling.^23^ The values obtained from the best-fit were *a* = 0.0014 and *b* = 0.34 (*R*^2^ = 0.98) for the sink condition and *a* = 0.41 and *b* = 0.51 (*R*^2^ = 0.99) for the confined volume release. Thus, μPLs provide a sustained release of DEX *via*, predominantly, a diffusion-controlled mechanism through the PLGA matrix within the first few weeks. In order to confirm this, particle degradation under the physiological condition was studied. As shown in Figure S2, on day 0, particles showed the characteristic well-defined squared morphology. On day 7, images documented a few signs of degradation on some μPLs. The degradation progressed over time, affecting mostly the inner, bottom part of some μPLs, while the edges of all the particles continued to be well defined for the entire first month of incubation. The gradual transition from a squared geometry to a round microparticle occurs only at the later stage (day 42). This would lead to conclude that, within the first weeks of incubation, only a modest portion of some μPLs is biodegraded.

After characterizing the *in vitro* drug release kinetics, a preliminary characterization of the μPL mechanical properties was performed using nanoindentation and dynamic mechanical analysis. Specifically, a small droplet (<1 μL) of a μPL suspension was deposited over a glass slide, dried overnight, and indented with a 200 μm-diameter truncated cone tip. Indentation force–displacement curves were derived as shown in Figure 2d, where the average value (line) and the corresponding standard deviation (shadowed area) are presented for three repetitions. From the slope of the force–displacement curves, an apparent modulus of 3.1 ± 0.9 MPa was calculated based on the classical Hertz theory of contact mechanics. In addition to this static characterization, dynamic testing was conducted to characterize the viscoelastic response and potential mechanical dampening behavior of μPLs. In this case, a small droplet of a μPL suspension was deposited over a glass slide and partially dried in a vacuum desiccator to create a thin particle layer. Then, a sinusoidal force was applied to the μPL layer, with a frequency of 5 Hz and increasing force amplitude (0.04, 0.08, and 0.12 N). The phase difference between the input (force) and output (deformation) was recorded over time to extract the phase difference parameter (tan δ), which is related to the mechanical damping of the system.^24^ This is shown in Figure 2e, giving a tan δ of ∼0.3. This dissipation value is characteristic of materials with high damping and shock absorbing properties.^25,26^

### 2.3 *In Vitro* Anti-inflammatory Effect of Dexamethasone-loaded MicroPlates (DEX-μPLs)

**Figure 3:**
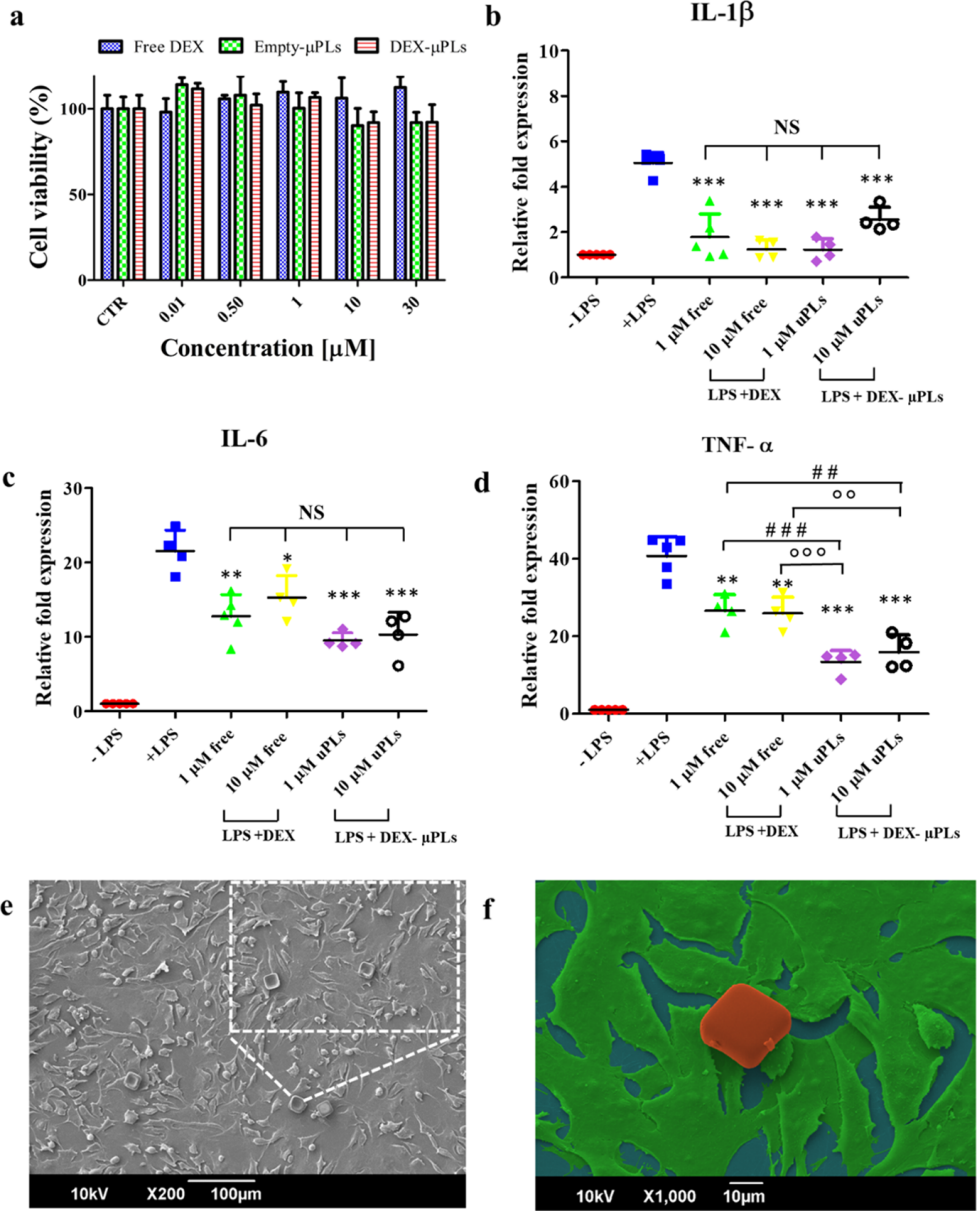
*In vitro* cytocompatibility and anti-inflammatory effect of DEX-loaded microPlates (DEX-μPLs). (a) ATDC5 cell viability upon incubation with free DEX, empty-μPLs, and DEX-μPLs. Statistical analysis *via* one-way ANOVA (GraphPad Prism 5) is provided in Table S3; (b–d) Expression levels of proinflammatory cytokines IL-1β, IL-6, and TNF-α for LPS-stimulated ATDC5 cells. (−LPS: no LPS and no μPLs; +LPS: LPS stimulation and no μPLs; DEX: LPS stimulation and free DEX at 1 and 10 μM; and DEX-μPLs: LPS treatment and DEX-μPLs at 1 and 10 μM). Results are presented as average ±SD (*n* ≥ 4). *p < 0.05, **p < 0.001, and ***p < 0.0001 were considered statistically significant as compared to the control (+LPS); ^##^*p* < 0.001 and ^###^*p* < 0.0001 were considered statistically significant as compared to 1 μM DEX; and ^oo^*p* < 0.001 and ^ooo^*p* < 0.0001 were considered statistically significant as compared to 10 μM DEX. The lack of a statistically significant difference between groups is indicated on the graphs as NS. Multiple comparisons were performed using, as the post hoc test, the Tukey’s significant difference (HSD) test; (e) 30° tilted view of a SEM image of ATDC5 cells incubated with μPLs. In the lateral inset, a magnified image shows cells interacting with μPLs; (f) False-color SEM image of a μPL (red) deposited and not internalized over a layer of ATDC5 cells (green).

In order to test potential toxicity effects, the proliferative activity of chondrogenic ATDC5 cells was measured after treatment with DEX, empty-μPLs, and DEX-μPLs (Figure 3a). Empty-μPL treatments were defined to contain the same polymeric amounts used for the treatment with DEX-μPLs. No toxicity effect was observed within a wide range of concentrations of free drug (up to 30 μM of DEX) and empty microparticles (up to 3 μPLs per cell). These results are in line with previous tests conducted by the authors on other cells, including primary bone marrow-derived monocytes.^17,27^ Also, the percentage of live and dead cells under different treatments was evaluated using two different techniques: trypan blue count and flow cytometry (FC) analysis. As shown in Figure S3 and the related table, both independent analyses returned ATCD5 viability values well over 90% for all tested experimental groups and at all tested concentrations.

Proinflammatory cytokines, produced primarily by synoviocytes and chondrocytes in the joint,^28^ promote the production of proteases that breakdown articular cartilage and collagen fibers.^29^ Thus, anti-inflammatory properties of the proposed drug delivery system were tested in ATDC5 cells treated with free DEX and DEX-μPLs, at 1 and 10 μM DEX concentrations, and then stimulated with LPS to trigger a rapid and robust proinflammatory response.^30−32^ DEX-μPLs and free DEX significantly (*p < 0.05, **p < 0.001, and ***p < 0.0001) reduced the expression of three inflammatory cytokines (IL-1β, IL-6, and TNF-α), as compared to the untreated samples that were just stimulated with LPS (+LPS) (Figure 3b–d). A DEX concentration of 1 μM was sufficient to inhibit the expression of all tested proinflammatory genes. The higher DEX concentration (10 μM) did not appear to significantly enhance the anti-inflammatory activity as compared to the lower dose. These data suggest that DEX-μPLs reduce the production of inflammatory cytokines by ATDC5 cells in response to potent proinflammatory stimuli. Similar results were previously reported by the authors on different cell types, including bone marrow-derived monocytes,^17,27^ confirming the anti-inflammatory properties of DEX-μPLs across multiple cell types relevant to PTOA. Also, as documented in Figure S4, the empty-μPLs did not induce any increase in proinflammatory cytokine expression at both tested particle concentrations, demonstrating that empty-μPLs lack any pro- or anti-inflammatory activity.

Confocal laser scanning microscopy was performed in order to observe the interaction between the fluorescently labeled μPLs and ATDC5 cells. Figures 3e,f, S5, and S6 confirm the absence of μPL internalization by ATDC5 cells and macrophages, suggesting that the particles would be retained extracellularly^33−35^ and therefore release DEX into the articular cartilage and intra-articular space to affect cells throughout the joint. Note that these particles are not expected to be phagocytosed but, instead, to biodegrade over time, releasing lactic and glycolic acid byproducts that enter the Kreb’s cycle to be eliminated as CO_2_ and water.^36^

### 2.4 *In Vivo* Pharmacokinetic, Biodistribution, and Confocal Microscopy Characterization of Cy5-μPLs

To assess the intra-articular retention time, the near infrared dye Cy5 was directly conjugated to the polymeric matrix of μPLs. Specifically, the carboxylic groups on the PLGA chain of μPLs were activated, *via* an 1-ethyl-3-(3-(dimethylamino)propyl)-carbodiimide (EDC)/*N*-hydroxysuccinimide (NHS) reaction, and then covalently coupled to the free amine group of the Cy5 molecule modified with a 1,8-diamino-3,6-dioxaoctane. The stability of the resulting Cy5-μPLs was evaluated upon particle incubation in PBS, at 37 ± 2 °C, up to 1 month (same duration of the *in vivo* experiment). As reported in Figure S7, after 24 h in PBS, 83.9 ± 1.6% of Cy5 molecules remained covalently bound to the μPL PLGA matrix.

**Figure 4:**
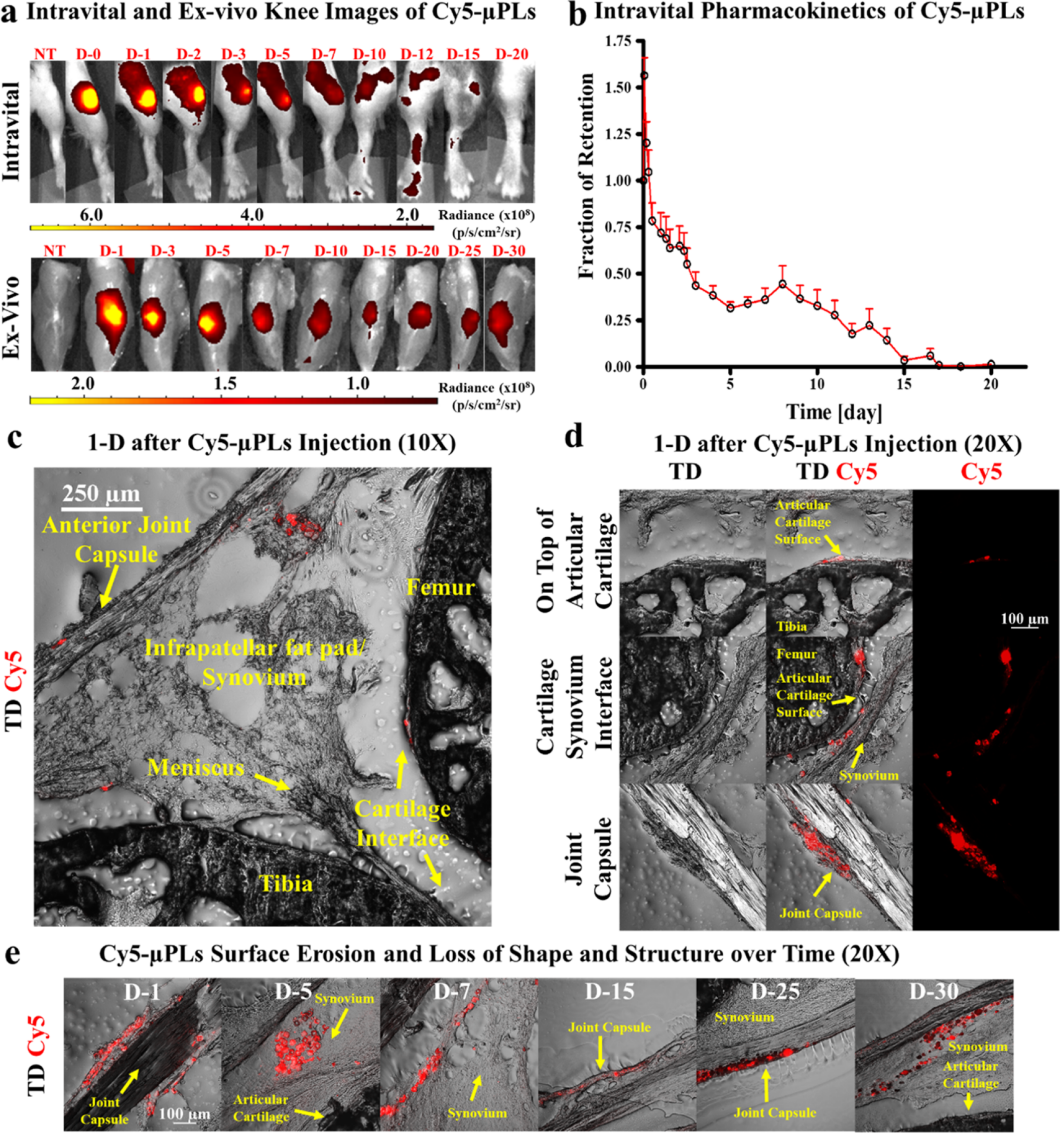
*In vivo* pharmacokinetic study of Cy5-conjugated μPLs (Cy5-μPLs) in a PTOA mouse model. (a) Representative pharmacokinetic time course intravital images (skin on) and *ex vivo* knee images (skin off) of Cy5-μPLs injected intra-articularly into PTOA mouse knee joints (D-#, where # represents days after intra-articular injection); (b) Intravital fraction of retention of Cy5-μPLs plotted as mean + standard error. Note = the initial uptick in fluorescence within the joints in the first couple of hours after injection is a result of loss of fluorophore self-quenching, which occurs due to high-density fluorophore conjugation onto the particles; (c) Anatomically labeled sagittal section of a mouse knee joint 1 day after intra-articular injection showing the Cy5-μPLs dispersed across the joint interacting and/or in close proximity to many different tissue types such as the cartilage, the infrapatellar fat pad and synovium, and the joint capsule; (d) Confocal microscopy imaging performed 1 day after intra-articular injection showing Cy5-μPLs located on top of the cartilage surface, near the cartilage/synovium interface, and the joint capsule. In all images, the scale bar = 100 μm; (e) Confocal microscopy imaging of Cy5-μPLs within the mouse knee joint taken at different time points after intra-articular injection. TD = transmission detector. NT = no treatment. For intravital imaging analysis, *n* = 4–24 limbs depending on the time point, that is, earlier time points had more animals included, and the sample size at the later time points was lower because some animals were taken down at earlier time points for *ex vivo* and confocal microscopy analysis. For *ex vivo* imaging analysis and confocal microscopy analysis, *n* = 2–4 limbs per time point.

A time course of intravital imaging, *ex vivo* imaging, organ biodistribution, and confocal microscopy analyses was performed following a single intra-articular injection of Cy5-μPLs into a cohort of mice with mechanically induced PTOA. The Cy5 fluorescence signal in the joint, associated with μPLs, was captured over 20 days *via* intravital imaging (Figure 4a, top row) and for the entire time course of 30 days *via* the more sensitive *ex vivo* analysis with the skin removed from the limb (Figure 4a, bottom row). This was translated into an intravital fraction of particle retention as a function of time, as plotted in Figure 4b. The initial, transient increase in fluorescence within the joints on the first day after injection (Figure 4b) is a result of loss of fluorophore self-quenching, which occurs due to high-density fluorophore conjugation into the particles. Confocal microscopy performed 1 day after injection showed the Cy5-μPLs dispersed across the entire knee joint (Figure 4c), reaching the femoral-tibial cartilage interface, the infrapatellar fat pad and synovium, and the joint capsule. Magnified images at 1 day after injection showed deposition of Cy5-μPLs on top of the articular cartilage surface, near the cartilage/synovium interface and the joint capsule (Figure 4d). Magnified images of Cy5-μPLs within the joint space at different time points document a heterogenous loss of Cy5 fluorescence over time, which could be correlated with surface particle erosion and distortion (Figures 4e, S8, and S9). At the later time points, Cy5-μPLs were seen mostly in the surrounding synovial, joint capsule, and soft-tissue structures rather than the cartilage interface (Figures 4e, S8, and S9). This may result from a combination of factors including greater degradation at the cartilage interface, the loading process pushing synovial fluid (and consequently μPLs) to the surrounding soft tissue structures, and histological sampling. Finally, biodistribution analyses showed the Cy5 fluorescence signal to be mostly localized within the knee joints (Figure S10).

### 2.5 Therapeutic Assessment of Dexamethasone-loaded MicroPlates (DEX-μPLs) in an Overload-Induced OA Mouse Model

**Figure 5:**
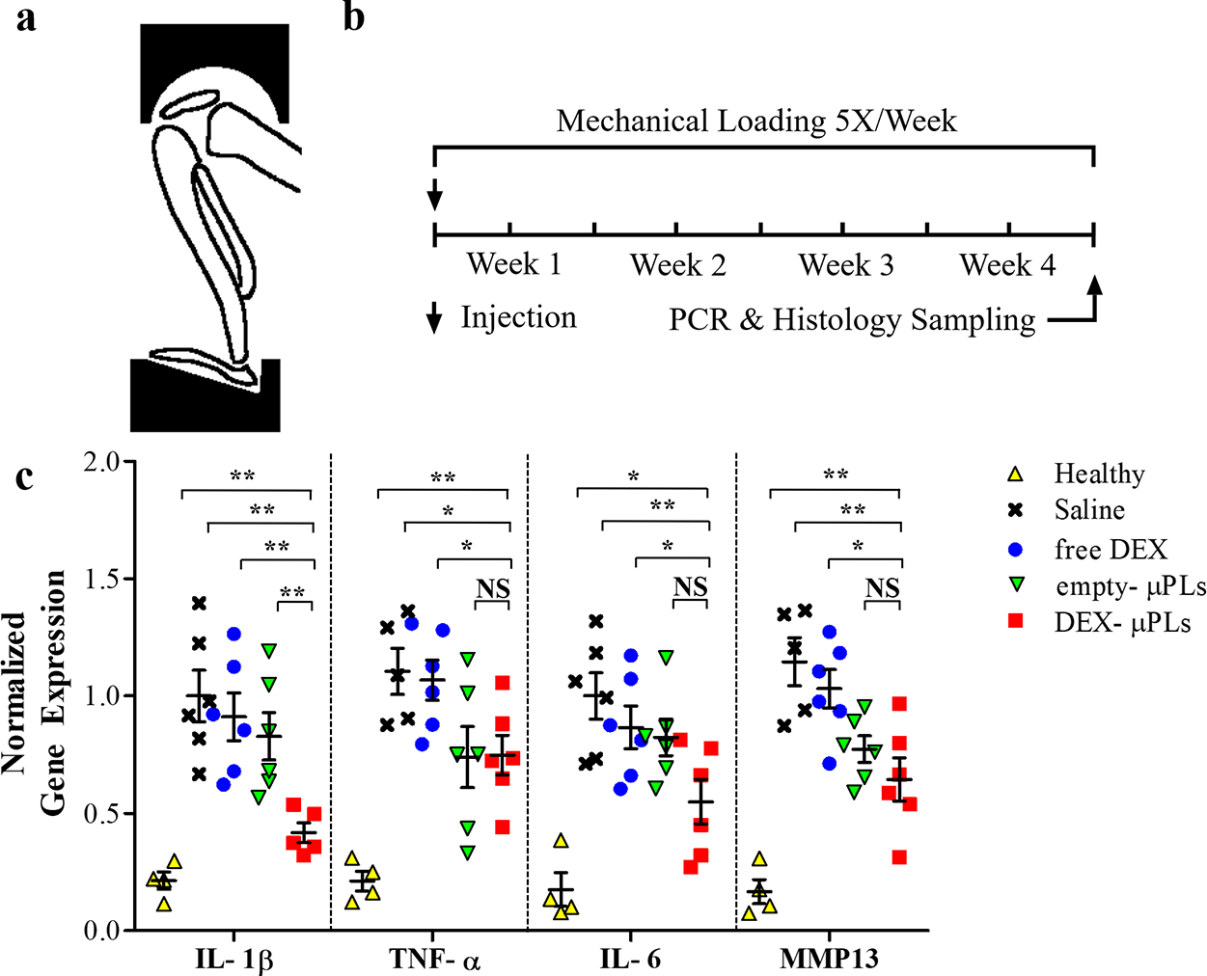
Proinflammatory gene expression in a PTOA model mouse. (a) Schematic of the loading fixture used in the mechanical loading of mouse knee joints to induce PTOA; (b) mechanical loading regimen; (c) *in vivo* expression of IL-1β, TNF-α, IL-6, and MMP-13 measured by TaqMan qPCR (for each treatment groups *n* = 6, while for the healthy group *n* = 4). Statistical analysis *via* one-way ANOVA (GraphPad Prism 8), corrected for multiple comparisons by controlling the false discovery rate with a two-stage, step-up Benjamini–Krieger–Yekutieli method: *p < 0.05 and **p < 0.01, while no significant differences are indicated on the graphs as NS. A full list of *p*-values is provided in Table. S4.

Next, a 4 week *in vivo* study was completed in a PTOA mouse model where the animals’ knees were placed in flexion and loaded in compression using a custom fitting, as depicted in Figure 5a. A single intra-articular dose of DEX (1 mg kg^–1^) was administered into each knee, as free DEX or DEX-μPLs, starting concurrently with mechanical loading. An identical quantity of μPLs without DEX was administered as a vehicle control. The DEX dose (animal weight adjusted) was chosen based on a dose that previously produced robust anti-inflammatory effects in rabbits^37^ and rats.^38^ Following 4 weeks of mechanical loading, qPCR was employed to assess expression of genes associated with PTOA progression on the combined tibial and femoral cartilage surfaces and synovial tissue (Figure 5b). Expression of proinflammatory cytokines IL-1β, IL-6, and TNF-α, in addition to matrix metalloproteinase-13 (MMP-13), was assessed. Note that MMP-13 is a primary driver of degradation of the key cartilage structural protein type II collagen.^39^ The data presented in Figure 5c, supported by the computed p-values listed in the Table S4, show that DEX-μPLs (red square) significantly reduced all genes assayed compared to untreated knees (black cross) (*p* values: IL-1β = 0.0013, TNF-α = 0.0222, IL-6 = 0.0083, and MMP-13 = 0.0053) and free DEX (blue circle) (*p* values: IL-1β = 0.0024, TNF-α = 0.0244, IL-6 = 0.0376, and MMP-13 = 0.0107). Interestingly, empty-μPLs (green triangle) did significantly reduce the expression of MMP-13 as compared to untreated knees (*p* value: 0.0086) and free DEX (*p* value: 0.0268). The marked reduction of expression for all inflammatory genes measured from a single dose of DEX-μPLs at the end of a rigorous 4 week (5 times per week) loading protocol implies a prolonged pharmacological effect of DEX-μPLs due to extended DEX availability within the intra-articular space. On the other hand, the therapeutic effect of free DEX on the cartilage structure and synovial health has been shown at comparable doses in rabbits to dissipate within 3 weeks of injection without formulation, further validating the observed extension of benefit from DEX-μPLs over free DEX.^40^

**Figure 6:**
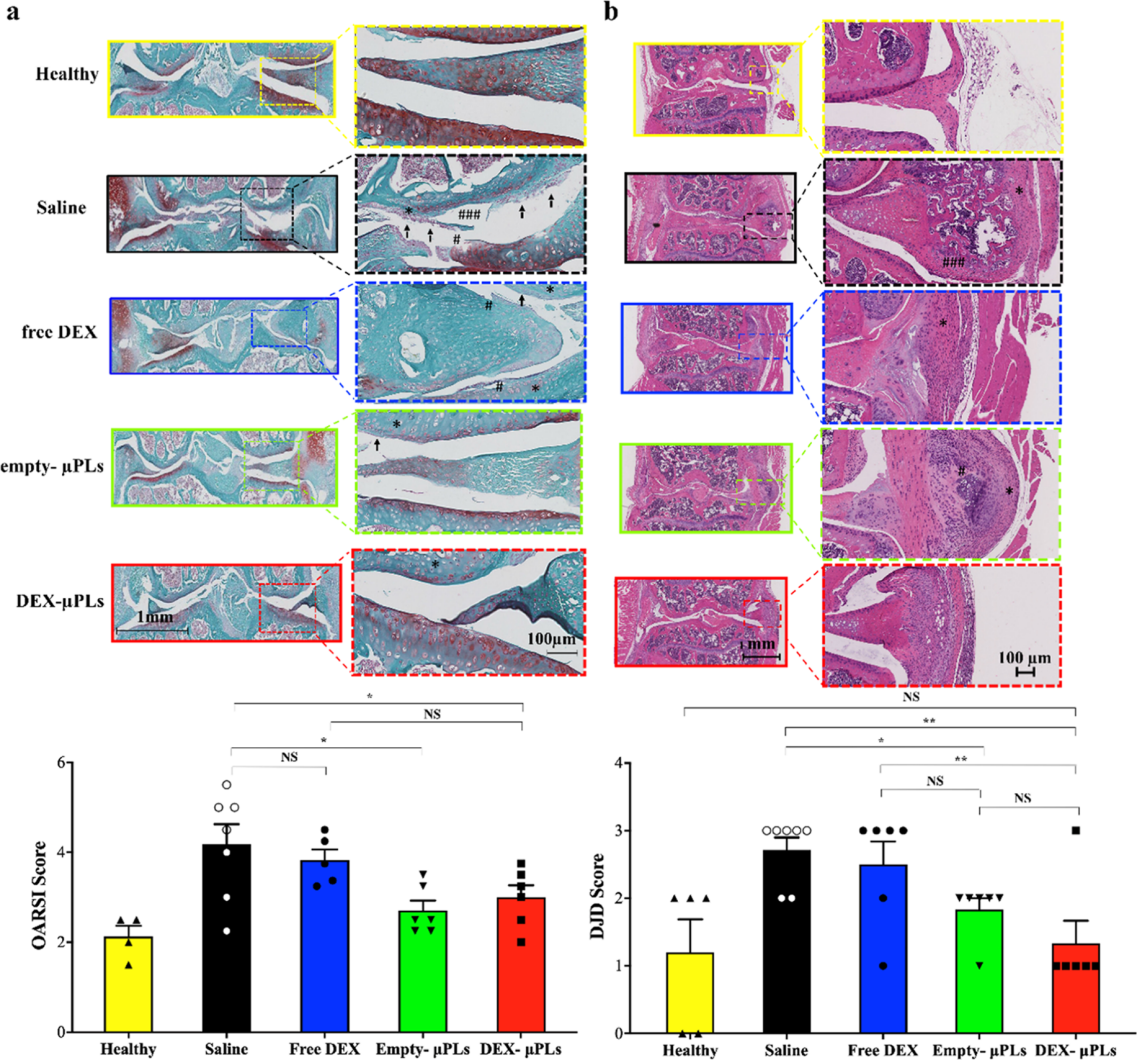
Safranin-O staining of joint sections (OARSI scoring) and H&E staining of joint sections in a PTOA mouse model (DJD scoring). (a) Representative safranin-O staining of the articular surface of the tibia and femur; insets show areas of interest under increased magnification (top) and treatment-blinded histological scoring by OARSI standards (bottom) (for each treatment groups, *n* = 6, while for the healthy group, *n* = 4). (Histology: arrows—cartilage erosion, #—cartilage fissures, *—low safranin-O staining; plotted data: *p < 0.05, no significant differences are indicated on the graphs as NS). Statistical significance *via* one-way ANOVA (GraphPad Prism 8), corrected for multiple comparisons by controlling the false discovery rate with a two-stage, step-up Benjamini–Krieger–Yekutieli method; all images are matched in the scale; (b) H&E staining of PTOA joints showing a representative slide for each treatment group (histology: #—mineralization and *—cellular infiltration and synovial membrane thickening); insets show higher magnification images of synovial tissue (top) and DJD scoring of the joint histology by a treatment-blinded pathologist (bottom, *p < 0.05, while no significant differences are indicated on the graphs as NS); statistical significance *via* one-way ANOVA (GraphPad Prism 8), corrected for multiple comparisons by controlling the false discovery rate with a two-stage, step-up Benjamini–Krieger–Yekutieli method, statistically; all images are matched in the scale. In the lower magnification images, scale bar = 1 mm, while in higher magnification images, scale bar = 100 μm (for each treatment groups, *n* = 6, while for the healthy group, *n* = 4).

Histology was also performed to assess the progression of PTOA in terms of structural deterioration of the articular cartilage and synovial response. Sections of each joint were stained with Safranin O and Fast Green to assess damage to the articular cartilage surface. Safranin O is a cationic dye that binds to proteoglycans, which are structural molecules depleted in the context of OA. This stain is used for grading the severity of OA by the Osteoarthritis Research Society International (OARSI) scoring method.^41,42^ A treatment-blinded histopathologist assessed Safranin O slides using the OARSI scale and found that DEX-μPLs (red bar) and empty-μPLs (green bar) significantly reduced the OARSI severity score compared to untreated joints (black bar) (*p* values: 0.037 and 0.01, respectively) (Figure 6a). Free DEX (blue bar) was not found to provide a significant difference in the OARSI score relative to untreated PTOA control knees.

Because OA is a total joint disease that involves remodeling of and crosstalk with the synovium, meniscus, and underlying subchondral bone,^29,43^ hematoxylin and eosin (H&E) staining was also used to appraise the overall joint health and more specifically inflammation, fibrosis, thickening, and mineralization in the surrounding soft tissues (synovium and meniscus). Treatment-blinded histopathological scoring was also completed with the degenerative joint disease (DJD) score, a joint scoring approach meant to complement the OARSI score by appraising joint health beyond cartilage integrity with regard to meniscal metaplasia/fragmentation, osteophyte formation, capsule fibrosis, and synovitis/synovial hyperplasia (Table S1). DJD scoring revealed that DEX-μPLs (red bar) and empty-μPLs (green bar) significantly improved the overall joint health over the untreated knees (black bar) (*p* value: 0.0027 and 0.0437, respectively) (Figure 6b). Also, DEX-μPLs (red bar) performed better than free DEX (blue bar) (*p*-value: 0.0012) in terms of DJD scoring (Figure 6b). The load-induced arthritis model in these studies is very aggressive and triggers a very intense onset of PTOA-associated changes in the knee joint, including synovial hyperplasia, osteophyte formation, chondrophyte formation, and ectopic mineralization in the synovium and meniscus. Joints treated with empty μPLs and DEX-μPLs showed milder and less mature chondrophyte formation, cellular infiltration, and synovial membrane thickening relative to the saline-treated animals. The saline-treated PTOA mice, in particular, showed much more advanced chondrophytes and osteophytes, showing signs of ectopic mineralization of the meniscus and synovium. These histological data confirm the significant therapeutic effect of DEX-μPLs as manifested by the reduction for the OARSI score and the DJD score as compared to the untreated and free-DEX-treated knees.

## 3 Discussion

Herein, we report that DEX-loaded μPLs with specific geometrical and mechanical properties could provide significant therapeutic benefits to PTOA joints. It is well documented that intra-articularly injected micron-sized particles, such as the 20 × 20 × 10 μm μPLs, would not be drained away from the joint *via* the synovial capillaries and lymphatics but would rather distribute within the synovial fluid and at the interface with the synovial lining and the articular cartilage.^14,44,45^ This was evident in the *in vivo* pharmacokinetic study where intravital, *ex vivo*, and confocal microscopy imaging detected Cy5-μPLs in the joint, particularly on top of the cartilage surfaces, near the infrapatellar fat pad/synovium and the joint capsule for up to 30 days. These are all sites of pathologic inflammation in arthropathies, including OA. Furthermore, the Cy5 signal was detected mostly in the knee joint with low levels seen in metabolic/clearance organs. This is consistent with the original objective as DEX-μPLs were designed to reside within the joint cavity and sustainably release DEX as a means to mitigate proinflammatory signals within the joint. Given the limited volume of the human synovial cavity (∼3 mL) and the release rates documented in Figure 2c under confined volume conditions, DEX could be sustainably released for several weeks to months from DEX-μPLs. Based on multiple reports, a strong anti-inflammatory activity results in pronounced differences in outcomes in the early stages of the disease, when inflammatory cytokines are mostly produced by macrophages and fibroblastic synoviocytes residing in the synovium.^2,46,47^ This could explain the significant reduction in the production of cytokines observed in the joints of mice treated with the DEX-μPLs as compared to free DEX, which has a half-life in the joint of only a few hours.^10^ Other publications corroborate the lack of protective effect of free DEX after 3–4 weeks and highlight the value of a platform that can continuously release DEX for a sustained period of time.^40,48^ Importantly, no statistically significant difference was observed between the untreated and free DEX-treated knees in cytokine production and MMP-13 expression (see Table S4) or for the two histological scoring systems applied (see the OARSI score in Figure 6a and DJD score in Figure 6b).

A notable advantage of DEX-μPLs over other microparticle drug delivery systems is the opportunity to precisely tailor the μPL size, geometry, surface, and mechanical properties during the top-down fabrication process. The size of μPLs can range from a few to several tens of micrometers; the fabrication templates can be altered to yield particles with a range of geometries; while the deformability can vary from a few kPa to tens of MPa depending on the amounts of PLGA used.^17,27^ Moreover, the surface physicochemical properties of μPLs can be modulated to facilitate their biochemical interaction with cartilage and HA in the synovial fluid, both of which are anionic. From the initial therapeutic findings, future work is merited to more rigorously study the interplay between the pharmacological efficacy of the DEX-loaded μPLs and the potential of mechanically tuning the μPLs to affect the rheological properties of the synovial fluid.

Finally, it is important to recall that studies in mice always have limitations when compared to an authentic human traumatic joint injury as the size scale and tissue content and architecture differ between species. However, it is important to highlight that the murine model utilized in the present study to create accelerated PTOA has the advantages of being applied to pathophysiologically relevant aged (6 month old) mice and of being based on rigorous cyclic mechanical loading^49^ rather than commonly used surgical or chemical OA induction procedures that lack clinical relevance or introduce confounding factors into therapeutic studies.

## 4 Conclusions

In this study, size-, shape-, and mechanically defined, monodispersed PLGA μPLs were applied for the intra-articular delivery of DEX. A top-down approach was used for μPL fabrication, obtaining consistently shaped particles with a dimension of 20 μm per side and a height of 10 μm. The anti-inflammatory molecule DEX was efficiently loaded into μPLs, and the resultant formulation achieved continuous release over a period of 10 days under infinite sink conditions and at least 1 month in biologically relevant, confined volumes. The DEX-μPLs reduced inflammatory gene expression both *in vitro* and *in vivo*. In a highly rigorous model of post traumatic overload-induced OA, a single intra-articular injection of Cy5-μPLs was detected in the joint space for up to 30 days. In the same animal model, a single intra-articular injection of DEX-μPLs holistically protected both the articular cartilage and the broader joint structure through 4 weeks of rigorous mechanical overloading of the joints. In sum, this work provides a proof of concept for the utility of shaped-defined and deformable μPLs in the protection against PTOA-associated joint deterioration.

## 5 Experimental Section

### 5.1 Materials

PDMS (Sylgard 184) and the elastomer were obtained from Dow Corning (Midland, Michigan, USA). PVA (*M*_w_ 31,000–50,000), PLGA (lactide/glycolide 50:50, *M*_w_ 38,000–54,000), dexamethasone acetate (DEX), EDC, NHS, acetonitrile, ATDC-5 cell line, MTT assay, bacterial lipopolysaccharides (LPS), paraformaldehyde (PFA), propidium iodide (PI), and trifluoroacetic acid (TFA) were purchased from Sigma-Aldrich (Saint Louis, Missouri, USA). High-glucose Dulbecco’s modified Eagle’s minimal essential medium (DMEM)/F-12 GlutaMAX, high-glucose Dulbecco’s modified Eagle’s minimal essential medium (DMEM) penicillin, streptomycin, and heat-inactivated fetal bovine serum (FBS) were purchased from Gibco (Invitrogen Corporation, Giuliano Milanese, Milan, Italy). The RAW 264.7 cell line was obtained from American Type Culture Collection (ATCC, LGC Standards, Teddington, UK). Cyanine5 NHS ester was purchased from Luminoprobe (Hunt Valley, MD, US). 1,8-Diamino-3,6-dioxaoctane and CURC were purchased from Alfa Aesar (Haverhill, Massachusetts, USA). C57BL/6 mice were purchased from Jackson Laboratory (Bar Harbor, Maine, USA). Mouse study TaqMan primers (IL-6: Mm01210732_g1, IL-1β: Mm00434228_m1, TNF-α: Mm00443258_m1, and MMP13: Mm00439491_m1) and reagents (as directed by standard protocols) were all purchased from Thermo Fisher Scientific (Waltham, Massachusetts, USA). All the reagents and other solvents were used as received.

### 5.2 μPL Fabrication Process

μPLs were synthetized using a top-down approach, as previously reported.^17,27^ Briefly, the silicon master template was developed using DLW, which allows transfer of a specific pattern on the silicon. In this case, the pattern is made out of square wells with a length (∼20 μm) and a height (∼10 μm) characteristic size and shape of the μPLs. The silicon pattern was replicated into PDMS and then into PVA templates, which showed the same arrays as the original silicon master template. The mixture of PLGA and drug (none in empty controls) was filled into the holes of PVA sacrificial templates. Particles were obtained after the purification from PVA solution. Each batch of particles for all *in vitro* and *in vivo* experiments was synthesized using 15 mg of PLGA, and when appropriate, DEX was dispersed in the polymeric mixture at 3.2 weight percentage (500 μg). For all *in vitro* studies, dose of DEX was adjusted based on total particle mass added, and amount of DEX relative PLGA mass was not adjusted as a variable in these studies. 1,8-Diamino-3,6-dioxaoctane (30 μL) was dissolved in dichloromethane (3 mL) and methanol (MeOH, 1.5 mL). Cyanine-5 NHS ester (0.25 equiv) was dissolved in dimethylformamide (200 mL) and added to the previous solution. A catalytic amount of triethylamine was added to the reaction which was left to stir for 16 h. The intended product was precipitated with cold diethyl ether. The product was washed three times with cold diethyl ether, obtaining a final product with a yield of 85%.^50^

For assessing *in vivo* μPLs pharmacokinetic and biodistribution, Cy5 was covalently conjugated to the surface of particles. This was required to ensure the stable attachment of the fluorophore to the particle. Briefly, purified μPLs were incubated with EDC/NHS at a molar ratio of 3:1 for 5 h under rotation at room temperature. After removing unlinked activators (5 min centrifugation at 1717*g*), activated μPLs (around 400,000 particles) were incubated overnight with Cy5 (50 μg). Free, unlinked Cy5 was removed with washing steps (5 min centrifugation at 1717*g*).

### 5.3 μPL Size, Size Distribution, and Shape

μPL size and shape were assessed *via* SEM (Elios Nanolab 650, FEI). Briefly, a drop of the sample was placed on a silicon template and sputtered with 10 nm of gold. Samples were analyzed with the instrument operating at an acceleration voltage of 5–15 keV. μPL concentration and size distribution were also measured through a Multisizer 4 COULTER particle counter (Beckman Coulter, USA). Morphology was examined by optical profilometry on a ZETA-20 optical profilometer (ZETA Instruments, San Jose, CA) equipped with a 100× objective, with a corresponding vertical resolution of 10 nm.

### 5.4 μPL Drug Loading and Release Characterization

DEX LE and entrapment efficiency (EE) of DEX-μPLs were evaluated as previously reported.^17,27^ Briefly, before the HPLC analysis, samples were lyophilized and dissolved in acetonitrile/H_2_O (1:1, v/v). HPLC (Agilent 1260 Infinity, Germany) was equipped with a 100 μL sample loop injector and a C18 column (4.6 × 250 mm, 5 μm particle size, Agilent, USA) for chromatographic separation. An isocratic condition (H_2_O + 0.1% (v/v) TFA/AcN + 0.1% (v/v) TFA, 50:50 v/v, 0.3 mL/min) was applied for DEX elution.
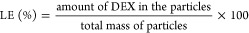
1
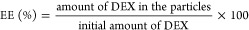
2

The kinetics of DEX release from μPLs was measured. To mimic an infinite sink condition, 200 μL of DEX-μPLs, corresponding to 10 μM DEX, was put into Slide-A-Lyzer MINI dialysis microtubes with a molecular cutoff of 10 kDa (Thermo Scientific) and then dialyzed against 4 L of PBS buffer (pH 7.4, 1×, 37 ± 2 °C). For each time point, three samples were collected and centrifuged (1717*g* for 5 min). Pellets were then dissolved in acetonitrile/H_2_O (1:1, v/v) and analyzed by HPLC. To evaluate the DEX release profile in a confined microenvironment, DEX-μPLs, corresponding to 10 μM DEX, were placed in three Eppendorf tubes in 500 μL of PBS buffer (pH 7.4, 1×, 37 ± 2 °C) under continuous rotation. For each time point, samples were collected and centrifuged (1717*g* for 5 min). The supernatant (100 μL) was added to 100 μL of acetonitrile, and the resultant samples were analyzed by HPLC. Pellets were then resuspended with 500 μL of fresh PBS buffer (pH 7.4, 1×). The experimental data were fitted to the two-phase Weibull equation model^23^
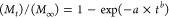
3where *M*_*t*_ and *M*_∞_ are the amounts of drug released at time *t* and equilibrium (infinite time), respectively. The variable *a* is a constant based on the system, and *b* is a constant based on the release kinetics. Values of *b* < 0.75 indicate that Fickian diffusion, not matrix degradation, is the dominant release mechanism.^23^

### 5.5 μPL Degradation Study

μPL matrix biodegradation was evaluated by SEM, as previously reported. Empty μPLs were incubated in PBS (pH = 7.4, 1×) under rotation at 37 ± 2 °C. At predetermined time points, an aliquot of the sample was collected and analyzed by SEM to investigate structural and morphological changes.

### 5.6 Cy5-μPL Stability and Release Profile

To assess Cy5-μPL stability over time, the release profile of Cy5 from μPLs was investigated. Cy5-μPLs were placed in three Eppendorf tubes in 500 μL of PBS buffer (pH 7.4, 1×, 37 ± 2 °C) under continuous rotation. For each time point, samples were collected and centrifuged (1717*g* for 5 min). Supernatants were dried and then resuspended in 200 μL of acetonitrile, and the resultant samples were analyzed using a μPL spectrophotometer with λ_ex_ 630 nm and λ_emi_ 660 nm (Tecan, Männedorf, Swiss). Pellets were then resuspended with 500 μL of fresh PBS buffer (pH 7.4, 1×).

### 5.7 Mechanical Characterization of μPLs

The apparent elastic modulus of μPLs was measured by flat punch microindentation tests. Small droplets (<1 μL) of a μPL suspension were deposited over a glass slide, covering an area of ∼5 mm^2^ with multiple particles and dried overnight. Microindentation was performed on a UNHT nanoindentation platform (Anton Paar) equipped with a 200 μm-diameter truncated cone tip. Load was applied at a rate of 20 mN/min until the maximum load of 3 mN. From the slope of the force–displacement curves, the modulus was calculated through the classical Hertzian equation *F* = 2*REh* where *F* is the applied force, *h* is the tip displacement, *R* is the tip radius, and *E* is the apparent elastic modulus. Three repetitions were conducted on different droplets.

The energy dissipation capability was characterized by dynamic mechanical analysis on a Q800 system (TA Instruments). Highly concentrated μPL suspensions were deposited on a glass slide and partially dried in a vacuum desiccator for 10 min to create a thin layer of μPLs. Then, the glass slide was transferred onto the bottom plate of a compressive clamp. A preload was applied gently, squeezing out excess water. A sinusoidal force was applied to the layer of μPLs with a frequency of 5 Hz and increasing amplitude (0.04, 0.08, and 0.12 N). The phase difference between the input (force) and output (deformation) was recorded as a function of the oscillation amplitude. The tangent of the phase difference angle, noted as tan δ, represents the ratio between dissipative and conservative energy during one oscillation and, as such, provides a measure of the damping capability of the material. Tests were conducted at 37 °C.

### 5.8 Toxicity of DEX-μPLs

ATDC5 cells were cultured at 37 °C in 5% CO_2_, in DMEM/F-12, GlutaMAX medium supplemented with 10% FBS, 1% penicillin/streptomycin. For the cell viability assay, cells at 80% confluence were seeded into 96-well plates at 10 × 10^3^ cells per well. After 24 h of incubation, cells were treated using different concentrations of free DEX, DEX-μPLs (namely, 0.01, 0.5, 1, 10, and 30 μM of DEX in all cases), or an equivalent number of empty μPLs matching the different DEX-μPL concentrations. The cytotoxicity was measured with the MTT assay (cell viability test). At the end of the designated incubation times, 5 mg/mL of MTT solution in PBS buffer was added to each well, and the cells were incubated for 4 h at 37 °C. The solubilized formazan product was quantified using a μPL spectrophotometer at 570 nm, using 650 nm as the reference wavelength (Tecan, Männedorf, Swiss). The percentage of cell viability was assessed according to the following equation
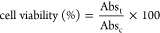
4where Abs_t_ and Abs_c_ were the absorbance of treated and control (untreated) cells, respectively.

### 5.9 Evaluation of the Percentage of Live/Dead Cells after Treatment with DEX-μPLs

2 × 10^5^ cells were seeded into each well of a 12-well plate, and after 24 h, cells were treated with free DEX, DEX-μPLs (namely, 0.01, 0.5, 1, 10, and 30 μM of DEX), or an equivalent number of empty-μPLs, for an additional 24 h. Then, the medium was removed from each well and collected. Cells were trypsinized, washed, and counted in the presence of trypan blue, which is excluded by live cells while entering dead cells. Cells were counted using a Countess II Automated Cell Counter (Thermo Fisher) after the addition of Trypan blue dye to discriminate live versus death cells. For the FC analysis, the collected cells were centrifuged and resuspended in PBS containing PI, according to the vendor’s instruction. After 15 min of incubation, each sample was analyzed using FACS ARIA (Becton Dickinson, USA). A cell population was selected setting a scatter gate that would exclude the negligible amounts of debris and aggregates.

### 5.10 Inflammatory Gene Expression Effects of DEX-μPLs *In Vitro*

To study the anti-inflammatory activity of DEX-μPLs in stimulated ATDC5, the expression levels of the three proinflammatory cytokines, TNF-α, IL-1β, and IL-6, were evaluated. Cells were seeded into six-well plates at 3 × 10^5^ cells per well for 24 h. After 5 h of preincubation with free DEX, DEX-μPLs at different concentrations (1 and 10 μM), or an equivalent number of empty μPLs matching the two DEX-μPL concentrations, cells were stimulated for 4 h with bacterial LPS (100 ng/mL). Then, RNA was extracted using an RNAeasy Plus Mini Kit (Qiagen) and quantified by NanoDrop2000 (Thermo Scientific, Waltham, Massachusetts, USA). Real-time RT-PCR was used to measure mRNA levels of inflammatory cytokines. For each condition, samples were run in triplicate. RT-PCR reactions were carried out using a Power SYBR Green RNA-to-CT 1-Step kit (Applied Biosystems) and using GAPDH expression as a housekeeping gene. Reactions were performed in a final volume of 20 μL. Oligonucleotide primer pairs were as follows: for GAPDH, 5′-GAACATCATCCCTGCATCCA-3′ and 5′-CCAGTGAGCTTCCCGTTCA-3′; for TNF-α, 5′-GGTGCCTATGTCTCAGCCTCTT-3′ and 5′-GCCATAGAACTGATGAGAGGGAG-3′; for IL-1β, 5′-TGGACCTTCCAGGATGAGGACA-3′ and 5′-GTTCATCTCGGAGCCTGTAGTG-3′; and for IL-6,5′-TACCACTTCACAAGTCGGAGGC-3′ and 5′-CTGCAAGTGCATCATCGTTGTTC-3′.

### 5.11 Cell/μPL Interaction as analyzed by Confocal Microscopy

To evaluate μPL cellular interactions, ATDC5 cells were seeded into an eight-chambered cover glass system (Lab Teck II, Thermo Scientific, USA) at 20 × 10^3^ cells per well and incubated for 24 h. The cells were then incubated overnight with CURC-μPLs (CURC loading used for fluorescence visualization purposes). The cells were fixed using 4% PFA, stained red using Alexa Fluor 488 Phalloidin (Thermo Fisher Scientific, USA), and nuclear stained using DAPI (Thermo Fisher Scientific, USA) following vendor instructions. Samples were analyzed using confocal microscopy (Nikon A1, Dexter, MI).

### 5.12 Cell/μPL Interaction as analyzed by SEM Analysis

To evaluate μPL cellular interactions within the ATDC5 cell line and phagocytic cell lines (RAW 264.7 macrophages), 20 × 10^4^ cells were seeded onto glass coverslips for 24 h. The cells were then incubated overnight with μPLs at a ratio of 1:4 (μPL/cells). Samples were fixed for 2 h in 2% glutaraldehyde in 0.1 M cacodylate buffer. After fixation, the samples were washed thrice with the same buffer and post fixed for 1 h in 1% osmium tetroxide in distilled water. After several washes with distilled water, the samples were subsequently dehydrated in a graded ethanol series, 1:1 ethanol/hexamethyldisilazane (HMDS) and 100% HMDS, followed by drying overnight in air. Dried samples were then mounted on stubs using silver conductive paint and coated with gold. SEM images were collected with SEM (Elios Nanolab 650, FEI) operating at an accelerating voltage between 5 and 15 keV.

### 5.13 *In Vivo* Pharmacokinetic and Biodistribution Study

An *in vivo* pharmacokinetic study was performed following the same loading and injection regimen as the mechanical-loading model described below (Section 5.13). Cy5-conjugated μPLs (Cy5-μPLs) were injected intra-articularly (as described in Section 5.13), and mice were imaged intravitally for Cy5 fluorescence over time using an IVIS Lumina III intravital imaging system (Caliper Life Sciences, Hopkinton, MA). For IVIS image analysis, regions of interest were drawn around both the right and the left knees. For each mouse knee, a preinjection reading (blank) was taken followed by a time 0 (*T*_0_) reading directly after intra-articular injection. The blank reading was used for background correction of all images of the same mouse knee at all time points, and dividing the radiance reading (at a specific time point) by the *T*_0_ radiance reading was used to calculate the “fraction of retention” at all later time points. Animals were euthanized at days 1, 3, 5, 7, 10, 15, 20, 25, and 30 postinjection along with control no treatment animals (NT). At takedown, the skin was removed from the legs, and the knees were endpoint-imaged “*ex vivo*” for Cy5 fluorescence which provides better sensitivity than intravital imaging. Organs were also harvested for Cy5 fluorescence biodistribution. After *ex vivo* imaging, excess muscle was removed, and legs were then snap frozen in liquid nitrogen and stored at −80 °C until cryosectioning. For intravital imaging analysis, *n* = 4–24 limbs depending on the time point, that is, earlier time points had more animals included as animals were taken down over time for *ex vivo* and confocal microscopy analysis. For *ex vivo* imaging analysis and confocal microscopy analysis, n = 2–4 limbs per time point.

### 5.14 Cryosection and Confocal Microscopy

Legs (stored at −80 °C) were embedded into the OCT freezing compound, cooled, and serial-sectioned in sagittal orientation until an adequate depth of the joint was reached. Cryosections at various depths along the joint were then sectioned at 20 μm thick, captured utilizing a commercially available polyvinylidene chloride film coated with synthetic rubber cement (http://section-lab.jp/), and placed on a slide. Slides were then fixed in 10% neutral buffered formalin for 5 min, cover-slipped with an aqua mount, and imaged on a Nikon Eclipse Ti inverted confocal microscope. Imaging settings were kept constant for imaging of all Cy5-μPLs-containing joint samples at each time point (*n* = 2–4 limbs per time point). TD = transmission detector.

### 5.15 *In Vivo* Mechanical Loading OA Model

Using a protocol approved by the Vanderbilt Institutional Animal Care and Use Committee, the PTOA model of noninvasive repetitive joint loading was adapted from previous studies^51,52^ to induce PTOA in the knee joints of mice using cyclical mechanical stress. The 28 C57BL/6 mice were aged to 6 months and subjected to a rigorous cyclic mechanical loading (on mice anesthetized with 3% isoflurane) at 9 N per load, 500 cycles per session, cycle lasting 2.5 s, 5 loading sessions per week, for 4 weeks using a TA ElectroForce 3100 (TA Instruments, New Castle, Delaware, USA).^49^ Cyclic loading is performed utilizing form-fitting insets, one that covers and stabilizes the kneecap and another that holds the ankle with both in a flexed position of 135°. Specifically, the mold for the kneecap is a half-sphere cavity measuring 5 mm in diameter, and the mold for the ankle is a cavity shaped as an equilateral triangular measuring 5 mm in diameter on each side. All loading is performed under anesthesia. Treatment groups each had six mice per group, while the unloaded, non-OA controls were four per group. Following synthesis, DEX-loaded and empty μPLs were dried and stored at +4 °C until the time of administration. μPLs were resuspended in PBS by 30 s of sonication immediately before intra-articular injection in 20 μL total volume. Intra-articular injections were administered medial to the patella and validated by repeated dye injections to ensure that articular delivery was successful. Dosing and rationale are described in the therapeutic assessment section. All treatment groups were administered a single time at the initiation of the mechanical loading.

### 5.16 Inflammatory Gene Expression Analysis from *In Vivo* PTOA Study

Gene expression was analyzed from homogenized cartilage and synovial tissues from one knee per animal with the primers and reagents listed in the Materials section. Under a surgical microscope, the cartilage of the tibial and femoral surfaces was excised by a scalpel and combined with synovial tissue at about a 1:1 cartilage/tissue mass ratio. Tissue homogenization was performed with 5 mm TissueLyser steel beads (Qiagen) in 2 mL tubes for 5 min at 30 Hz, and RNA was collected using the RNeasy mini-prep kit (Qiagen). The iScript cDNA RT kit (Bio Rad) was used for cDNA production. TaqMan qPCR primers and reagents were used as directed by standard protocols from Thermo Fisher Scientific (Waltham, Massachusetts, USA). Expression was normalized to both GAPDH and ACTINB housekeeping genes before mRNA expression was normalized against the group indicated in each figure caption and quantified using the 2^–ΔΔCT^ method (IL-6: Mm01210732_g1, IL-1β: Mm00434228_m1, TNF-α: Mm00443258_m1, and MMP13: Mm00439491_m1).

### 5.17 Histology

Stifles were fixed in 10% neutral buffered formalin and decalcified in Immunocal for 72 h (StatLab, McKinney, TX). All tissue handling for histopathology was performed in the Vanderbilt Translational Pathology Shared Resource by certified histotechnicians. Fixed tissues were routinely processed using a standard 8 h processing cycle of graded alcohols, xylenes, and paraffin wax, embedded, sectioned at 5 μm, floated on a water bath, and mounted on positively charged glass slides. H&E staining was performed on the Gemini autostainer (Thermo Fisher Scientific, Waltham, MA). Safranin O staining was performed by hand using a kit (StatLab). Stifle joints were evaluated by H&E and safranin O in at least two serial mid-frontal (coronal) sections. Each mouse knee underwent serial anterior coronal sections every 50 μm from the point where there was disappearance of the patella to the point where there was loss of femoral condyles. All these sections (>10 sections per joint) were assessed by the treatment-blinded pathologist from which two representative sections were selected and used for scoring. This down-selection approach by a blinded third party was performed to reduce noise in scoring while appropriately representing each joint. This histopathologic section screening and scoring were conducted by a board-certified veterinary pathologist under treatment-blinded conditions.^42^ OARSI scores (0–6 semiquantitative scale) were provided for the medial tibial plateau and lateral tibial plateau.^53^ Simultaneously, a more holistic scoring method, the DJD scale (0–3 semiquantitative), was also used to supplement OARSI scoring of cartilage integrity with criteria appraising tissue inflammation, changes in bone morphology, and other signs of joint deterioration as defined in the included criteria (Table S1).^53^ All the scoring was carried out by a blinded pathologist, according to the criteria laid out in Table S1. The most relevant features in the scoring were cartilage degeneration, meniscal metaplasia, subchondral osteosclerosis, synovial hyperplasia and inflammation, and osteophyte formation. For both analyses, the histopathologist had access to all sections from each joint and chose a section representative of the individual joint for scoring while still blinded to the treatment group.

## Supporting Information Available

Safranin O scoring of tibial plateau cartilage degeneration and H&E scoring of the overall stifle DJD; μPL volumes prior and after lyophilization *via* optical profilometric analysis; list of *p* values for experimental groups of Figure 3a; list of *p*-values for all the experimental groups of Figure 5; profilometric analysis of hydrated and lyophilized μPLs; particle degradation; determination of live/dead cells after dexamethasone-loaded microPlates (DEX-μPLs); *in vitro* effect of empty μPLs; ATDC5 cell interaction with μPLs; RAW 264.7 macrophages interacting with μPLs; Cy5-μPL stability; 20× images of Cy5-μPLs within a mouse model of PTOA at multiple time points; magnified images of Cy5-μPLs within a mouse model of PTOA at multiple time points; and Cy5-μPL *ex vivo* organ biodistribution in a mouse model of PTOA at multiple time points (PDF)

## References

[ref1] Loeser R. F.; Collins J. A.; Diekman B. O. Ageing and the Pathogenesis of Osteoarthritis. Nat. Rev. Rheumatol. 2016, 12, 412–420. 10.1038/nrrheum.2016.65. 27192932 PMC4938009

[ref2] Martel-Pelletier J.; Barr A. J.; Cicuttini F. M.; Conaghan P. G.; Cooper C.; Goldring M. B.; Goldring S. R.; Jones G.; Teichtahl A. J.; Pelletier J.-P. Osteoarthritis. Nat. Rev. Dis. Primers 2016, 2, 16072 10.1038/nrdp.2016.72. 27734845

[ref3] Little C. B.; Hunter D. J. Post-Traumatic Osteoarthritis: From Mouse Models to Clinical Trials. Nat. Rev. Rheumatol. 2013, 9, 485 10.1038/nrrheum.2013.72. 23689231

[ref4] Grodzinsky A. J.; Wang Y.; Kakar S.; Vrahas M. S.; Evans C. H. Intra-Articular Dexamethasone to Inhibit the Development of Post-Traumatic Osteoarthritis. J. Orthop. Res. 2017, 35, 406–411. 10.1002/jor.23295. 27176565 PMC5604325

[ref5] Olson S. A.; Horne P.; Furman B.; Huebner J.; Al-Rashid M.; Kraus V. B.; Guilak F. The Role of Cytokines in Posttraumatic Arthritis. J. Am. Acad. Orthop. Surg. 2014, 22, 29–37. 10.5435/jaaos-22-01-29. 24382877

[ref6] Bradley J. D.; Brandt K. D.; Katz B. P.; Kalasinski L. A.; Ryan S. I. Comparison of an Antiinflammatory Dose of Ibuprofen, an Analgesic Dose of Ibuprofen, and Acetaminophen in the Treatment of Patients with Osteoarthritis of the Knee. N. Engl. J. Med. 1991, 325, 87–91. 10.1056/nejm199107113250203. 2052056

[ref7] Kolasinski S. L.; Neogi T.; Hochberg M. C.; Oatis C.; Guyatt G.; Block J.; Callahan L.; Copenhaver C.; Dodge C.; Felson D. 2019 American College of Rheumatology/Arthritis Foundation Guideline for the Management of Osteoarthritis of the Hand, Hip, and Knee. Arthritis Rheumatol. 2020, 72, 220–233. 10.1002/art.41142. 31908163 PMC10518852

[ref8] McAlindon T. E.; Bannuru R. R.; Sullivan M. C.; Arden N. K.; Berenbaum F.; Bierma-Zeinstra S. M.; Hawker G. A.; Henrotin Y.; Hunter D. J.; Kawaguchi H.; Kwoh K.; Lohmander S.; Rannou F.; Roos E. M.; Underwood M. Oarsi Guidelines for the Non-Surgical Management of Knee Osteoarthritis. Osteoarthritis Cartilage 2014, 22, 363–388. 10.1016/j.joca.2014.01.003. 24462672

[ref9] Ayhan E.; Kesmezacar H.; Akgun I. Intraarticular Injections (Corticosteroid, Hyaluronic Acid, Platelet Rich Plasma) for the Knee Osteoarthritis. World J. Orthoped. 2014, 5, 351 10.5312/wjo.v5.i3.351. PMC4095029 25035839

[ref10] Larsen C.; Østergaard J.; Larsen S. W.; Jensen H.; Jacobsen S.; Lindegaard C.; Andersen P. H. Intra-Articular Depot Formulation Principles: Role in the Management of Postoperative Pain and Arthritic Disorders. J. Pharmaceut. Sci. 2008, 97, 4622–4654. 10.1002/jps.21346. 18306275

[ref11] Wu P.; Grainger D. W. Drug/Device Combinations for Local Drug Therapies and Infection Prophylaxis. Biomaterials 2006, 27, 2450–2467. 10.1016/j.biomaterials.2005.11.031. 16337266

[ref12] Gao W.; Chen Y.; Zhang Y.; Zhang Q.; Zhang L. Nanoparticle-Based Local Antimicrobial Drug Delivery. Adv. Drug Deliv. Rev. 2018, 127, 46–57. 10.1016/j.addr.2017.09.015. 28939377 PMC5860926

[ref13] Bodick N.; Lufkin J.; Willwerth C.; Kumar A.; Bolognese J.; Schoonmaker C.; Ballal R.; Hunter D.; Clayman M. An Intra-Articular, Extended-Release Formulation of Triamcinolone Acetonide Prolongs and Amplifies Analgesic Effect in Patients with Osteoarthritis of the Knee: A Randomized Clinical Trial. J. Bone Joint Surg. 2015, 97, 877–888. 10.2106/jbjs.n.00918. 26041848

[ref14] Maudens P.; Jordan O.; Allémann E. Recent Advances in Intra-Articular Drug Delivery Systems for Osteoarthritis Therapy. Drug discovery today 2018, 23, 1761–1775. 10.1016/j.drudis.2018.05.023. 29792929

[ref15] Piuzzi N. S.; Midura R. J.; Muschler G. F.; Hascall V. C. Intra-Articular Hyaluronan Injections for the Treatment of Osteoarthritis: Perspective for the Mechanism of Action. Ther. Adv. Musculoskeletal Dis. 2018, 10, 55–57. 10.1177/1759720x17752038. PMC5784477 29387178

[ref16] Stefani R. M.; Lee A. J.; Tan A. R.; Halder S. S.; Hu Y.; Guo X. E.; Stoker A. M.; Ateshian G. A.; Marra K. G.; Cook J. L.; Hung C. T. Sustained Low-Dose Dexamethasone Delivery Via a Plga Microsphere-Embedded Agarose Implant for Enhanced Osteochondral Repair. Acta Biomater. 2020, 102, 326–340. 10.1016/j.actbio.2019.11.052. 31805408 PMC6956850

[ref17] Di Francesco M.; Primavera R.; Romanelli D.; Palomba R.; Pereira R. C.; Catelani T.; Celia C.; Di Marzio L.; Fresta M.; Di Mascolo D.; Decuzzi P. Hierarchical Microplates as Drug Depots with Controlled Geometry, Rigidity, and Therapeutic Efficacy. ACS Appl. Mater. Interfaces 2018, 10, 9280–9289. 10.1021/acsami.7b19136. 29481038

[ref18] Han F. Y.; Thurecht K. J.; Whittaker A. K.; Smith M. T. Bioerodable Plga-Based Microparticles for Producing Sustained-Release Drug Formulations and Strategies for Improving Drug Loading. Front. Pharmacol. 2016, 7, 185 10.3389/fphar.2016.00185. 27445821 PMC4923250

[ref19] Dawes G. J. S.; Fratila-Apachitei L. E.; Necula B. S.; Apachitei I.; Witkamp G. J.; Duszczyk J. Release of Plga–Encapsulated Dexamethasone from Microsphere Loaded Porous Surfaces. J. Mater. Sci.: Mater. Med. 2010, 21, 215–221. 10.1007/s10856-009-3846-6. 19669866 PMC2805798

[ref20] Kraus V. B.; Stabler T. V.; Kong S. Y.; Varju G.; McDaniel G. Measurement of Synovial Fluid Volume Using Urea. Osteoarthritis Cartilage 2007, 15, 1217–1220. 10.1016/j.joca.2007.03.017. 17507255 PMC2034527

[ref21] Fredenberg S.; Wahlgren M.; Reslow M.; Axelsson A. The Mechanisms of Drug Release in Poly (Lactic-Co-Glycolic Acid)-Based Drug Delivery Systems—a Review. Int. J. Pharm. 2011, 415, 34–52. 10.1016/j.ijpharm.2011.05.049. 21640806

[ref22] Li C.; Wang B.; Liu X.; Pan Z.; Liu C.; Ma H.; Liu X.; Liu L.; Jiang C. The Dosage Effects of Dexamethasone on Osteogenic Activity Andbiocompatibility of Poly (Lactic-Co-Glycolic Acid)/Hydroxyapatite Nanofibers. Artif. Cells, Nanomed., Biotechnol. 2019, 47, 1823–1832. 10.1080/21691401.2019.1609007. 31066304

[ref23] Papadopoulou V.; Kosmidis K.; Vlachou M.; Macheras P. On the Use of the Weibull Function for the Discernment of Drug Release Mechanisms. Int. J. Pharm. 2006, 309, 44–50. 10.1016/j.ijpharm.2005.10.044. 16376033

[ref24] Coluccino L.; Peres C.; Gottardi R.; Bianchini P.; Diaspro A.; Ceseracciu L. Anisotropy in the Viscoelastic Response of Knee Meniscus Cartilage. J. Appl. Biomater. Funct. Mater. 2017, 15, 77–83. 10.5301/jabfm.5000319. 27647392

[ref25] Li Z.; Lu X.; Tao G.; Guo J.; Jiang H. Damping Elastomer with Broad Temperature Range Based on Irregular Networks Formed by End-Linking of Hydroxyl-Terminated Poly (Dimethylsiloxane). Polym. Eng. Sci. 2016, 56, 97–102. 10.1002/pen.24196.

[ref26] Chen S.; Wang Q.; Wang T.; Pei X. Preparation, Damping and Thermal Properties of Potassium Titanate Whiskers Filled Castor Oil-Based Polyurethane/Epoxy Interpenetrating Polymer Network Composites. Mater. Des. 2011, 32, 803–807. 10.1016/j.matdes.2010.07.021.

[ref27] Di Francesco M.; Primavera R.; Summa M.; Pannuzzo M.; Di Francesco V.; Di Mascolo D.; Bertorelli R.; Decuzzi P. Engineering Shape-Defined Plga Microplates for the Sustained Release of Anti-Inflammatory Molecules. J. Controlled Release 2020, 319, 201–212. 10.1016/j.jconrel.2019.12.039. 31899267

[ref28] Livshits G.; Kalinkovich A. Hierarchical, Imbalanced Pro-Inflammatory Cytokine Networks Govern the Pathogenesis of Chronic Arthropathies. Osteoarthritis Cartilage 2018, 26, 7–17. 10.1016/j.joca.2017.10.013. 29074297

[ref29] Loeser R. F.; Goldring S. R.; Scanzello C. R.; Goldring M. B. Osteoarthritis: A Disease of the Joint as an Organ. Arthritis Rheum. 2012, 64, 1697 10.1002/art.34453. 22392533 PMC3366018

[ref30] Huang Z.; Kraus V. B. Does Lipopolysaccharide-Mediated Inflammation Have a Role in Oa?. Nat. Rev. Rheumatol. 2016, 12, 123 10.1038/nrrheum.2015.158. 26656661 PMC4930555

[ref31] Yao Y.; Wang Y. Atdc5: An Excellent in Vitro Model Cell Line for Skeletal Development. J. Cell. Biochem. 2013, 114, 1223–1229. 10.1002/jcb.24467. 23192741

[ref32] Jin H.; Zhang H.; Ma T.; Lan H.; Feng S.; Zhu H.; Ji Y. Resveratrol Protects Murine Chondrogenic Atdc5 Cells against Lps-Induced Inflammatory Injury through up-Regulating Mir-146b. Cell. Physiol. Biochem. 2018, 47, 972–980. 10.1159/000490141. 29843156

[ref33] Champion J. A.; Walker A.; Mitragotri S. Role of Particle Size in Phagocytosis of Polymeric Microspheres. Pharmaceut. Res. 2008, 25, 1815–1821. 10.1007/s11095-008-9562-y. PMC2793372 18373181

[ref34] Champion J. A.; Mitragotri S. Shape Induced Inhibition of Phagocytosis of Polymer Particles. Pharmaceut. Res. 2009, 26, 244–249. 10.1007/s11095-008-9626-z. PMC2810499 18548338

[ref35] Paul D.; Achouri S.; Yoon Y.-Z.; Herre J.; Bryant C. E.; Cicuta P. Phagocytosis Dynamics Depends on Target Shape. Biophys. J. 2013, 105, 1143–1150. 10.1016/j.bpj.2013.07.036. 24010657 PMC3762343

[ref36] Elmowafy E. M.; Tiboni M.; Soliman M. E. Biocompatibility, Biodegradation and Biomedical Applications of Poly(Lactic Acid)/Poly(Lactic-Co-Glycolic Acid) Micro and Nanoparticles. J. Pharm. Invest. 2019, 49, 347–380. 10.1007/s40005-019-00439-x.

[ref37] Kydd A. S.; Reno C.; Sorbetti J. M.; Hart D. A. Influence of a Single Systemic Corticosteroid Injection on Mrna Levels for a Subset of Genes in Connective Tissues of the Rabbit Knee: A Comparison of Steroid Types and Effect of Skeletal Maturity. J. Rheumatol. 2005, 32, 307–319. 15693093

[ref38] Elron-Gross I.; Glucksam Y.; Margalit R. Liposomal Dexamethasone–Diclofenac Combinations for Local Osteoarthritis Treatment. Int. J. Pharm. 2009, 376, 84–91. 10.1016/j.ijpharm.2009.04.025. 19409466

[ref39] Mitchell P. G.; Magna H. A.; Reeves L. M.; Lopresti-Morrow L. L.; Yocum S. A.; Rosner P. J.; Geoghegan K. F.; Hambor J. E. Cloning, Expression, and Type Ii Collagenolytic Activity of Matrix Metalloproteinase-13 from Human Osteoarthritic Cartilage. J. Clin. Invest. 1996, 97, 761–768. 10.1172/jci118475. 8609233 PMC507114

[ref40] Bajpayee A. G.; Rodolfo E.; De la Vega R.; Scheu M.; Varady N.; Yannatos I.; Brown L.; Krishnan Y.; Fitzsimons T.; Bhattacharya P.; Frank E.; Grodzinsky A.; Porter R. Sustained Intra-Cartilage Delivery of Low Dose Dexamethasone Using a Cationic Carrier for Treatment of Post Traumatic Osteoarthritis. Eur. Cell. Mater. 2017, 34, 341 10.22203/ecm.v034a21. 29205258 PMC5744663

[ref41] McIlwraith C. W.; Frisbie D. D.; Kawcak C. E.; Fuller C. J.; Hurtig M.; Cruz A. The Oarsi Histopathology Initiative–Recommendations for Histological Assessments of Osteoarthritis in the Horse. Osteoarthritis Cartilage 2010, 18, S93–S105. 10.1016/j.joca.2010.05.031. 20864027

[ref42] Bolon B.; Stolina M.; King C.; Middleton S.; Gasser J.; Zack D.; Feige U. Rodent Preclinical Models for Developing Novel Antiarthritic Molecules: Comparative Biology and Preferred Methods for Evaluating Efficacy. J. Biomed. Biotechnol. 2011, 2011, 569068 10.1155/2011/569068. 21253435 PMC3022224

[ref43] Goldring M. B.; Goldring S. R. Articular Cartilage and Subchondral Bone in the Pathogenesis of Osteoarthritis. Ann. N.Y. Acad. Sci. 2010, 1192, 230–237. 10.1111/j.1749-6632.2009.05240.x. 20392241

[ref44] Brown S.; Kumar S.; Sharma B. Intra-Articular Targeting of Nanomaterials for the Treatment of Osteoarthritis. Acta Biomater. 2019, 93, 239–257. 10.1016/j.actbio.2019.03.010. 30862551 PMC6615949

[ref45] Bajpayee A. G.; Grodzinsky A. J. Cartilage-Targeting Drug Delivery: Can Electrostatic Interactions Help?. Nat. Rev. Rheumatol. 2017, 13, 183 10.1038/nrrheum.2016.210. 28202920

[ref46] Lopes E. B. P.; Filiberti A.; Husain S. A.; Humphrey M. B. Immune Contributions to Osteoarthritis. Curr. Osteoporos. Rep. 2017, 15, 593–600. 10.1007/s11914-017-0411-y. 29098574

[ref47] Mort J. S.; Billington C. J. Articular Cartilage and Changes in Arthritis: Matrix Degradation. Arthritis Res. Ther. 2001, 3, 337 10.1186/ar325. PMC128908 11714387

[ref48] Bajpayee A. G.; Wong C. R.; Bawendi M. G.; Frank E. H.; Grodzinsky A. J. Avidin as a Model for Charge Driven Transport into Cartilage and Drug Delivery for Treating Early Stage Post-Traumatic Osteoarthritis. Biomaterials 2014, 35, 538–549. 10.1016/j.biomaterials.2013.09.091. 24120044 PMC3863604

[ref49] Ko F. C.; Dragomir C.; Plumb D. A.; Goldring S. R.; Wright T. M.; Goldring M. B.; van der Meulen M. C. H. In Vivo Cyclic Compression Causes Cartilage Degeneration and Subchondral Bone Changes in Mouse Tibiae. Arthritis Rheum. 2013, 65, 1569–1578. 10.1002/art.37906. 23436303 PMC3672344

[ref50] Ferreira M.; Rizzuti I. F.; Palange A. L.; Barbato M. G.; Di Francesco V.; Di Francesco M.; Decuzzi P. Optimizing the Pharmacological Properties of Discoidal Polymeric Nanoconstructs against Triple-Negative Breast Cancer Cells. Front. Bioeng. Biotechnol. 2020, 8, 5 10.3389/fbioe.2020.00005. 32140459 PMC7042398

[ref51] Poulet B.; Hamilton R. W.; Shefelbine S.; Pitsillides A. A. Characterizing a Novel and Adjustable Noninvasive Murine Joint Loading Model. Arthritis Rheum. 2011, 63, 137–147. 10.1002/art.27765. 20882669

[ref52] Cho H.; Pinkhassik E.; David V.; Stuart J. M.; Hasty K. A. Detection of Early Cartilage Damage Using Targeted Nanosomes in a Post-Traumatic Osteoarthritis Mouse Model. Nanomedicine 2015, 11, 939–946. 10.1016/j.nano.2015.01.011. 25680539

[ref53] Glasson S. S.; Chambers M. G.; Van Den Berg W. B.; Little C. B. The Oarsi Histopathology Initiative - Recommendations for Histological Assessments of Osteoarthritis in the Mouse. Osteoarthritis Cartilage 2010, 18, S17–S23. 10.1016/j.joca.2010.05.025. 20864019

