# Supplementary material for: Shape-Defined microPlates for the Sustained Intra-articular Release of Dexamethasone in the Management of Overload-Induced Osteoarthritis
Source: ACS Appl Mater Interfaces. 2021 Jul 1;13(27):31379–92. doi: 10.1021/acsami.1c02082 (PMC8283751; doi:10.1021/acsami.1c02082)
Supplement: Supplementary file 1 — am1c02082_si_001.pdf [file am1c02082_si_001.pdf]

SUPPORTING INFORMATION

SHAPE-DEFINED MICROPLATES FOR THE SUSTAINED INTRAARTICULAR RELEASE OF DEXAMETHASONE IN THE MANAGEMENT OF OVERLOAD-INDUCED OSTEOARTHRITIS

*Martina Di Francesco[1, ♣], Sean K. Bedingfield[2, ♣], Valentina Di Francesco[1,3], Juan M. Colazo[2], Fang Yu[2], Luca Ceseracciu[4], Elena Bellotti[1], Daniele Di Mascolo[1], Miguel Ferreira[1], Lauren E. Himmel[5], Craig Duvall[2,#], and Paolo Decuzzi [1,# *]*

1. Laboratory of Nanotechnology for Precision Medicine, Istituto Italiano di Tecnologia, Via Morego 30, Genoa 16163, Italy
2. Department of Biomedical Engineering, Vanderbilt University, Nashville 37212, TN, United States
3. Department of Informatics, Bioengineering, Robotics and System Engineering, University of Genoa, Via Opera Pia 13, Genoa 16145, Italy.
4. Materials Characterization Facility, Istituto Italiano di Tecnologia, Via Morego 30, 16163 Genova, Italy
5. Department of Pathology, Microbiology and Immunology, Vanderbilt University Medical Center, Nashville 37212, TN, United States

♣ Martina Di Francesco, and Sean K. Bedingfield contributed equally to this work.

Craig Duvall and Paolo Decuzzi share the senior authorship

* Corresponding author: Paolo Decuzzi, PhD, paolo.decuzzi@iit.it

Number of pages: 13

Number of figures: 10

Number of tables: 4

SUPPORTING METHODS

Degenerative Joint Disease (DJD) scoring system. The Degenerative Joint Disease (DJD) scoring system was implemented to complement the OARSI scoring by assessing the joint holistically, scoring also for soft tissue and meniscal markers of PTOA advancement. [1]All scoring was done by a blinded pathologist, according to the criteria laid out in **Table S1**.

Severity Score	OARSI Scale	DJD Scale
0	Normal	Within normal limits: mild DJD as a feature of age-related change (e.g. articular cartilage attenuation and proteoglycan loss)
1	Loss of SO staining, thinning of articular cartilage; no defects	Moderate: articular cartilage degeneration with beginning of secondary pathology, including synovitis, joint capsule fibrosis, meniscal metaplasia
2	1 + fibrillation or pyknotic articular chondrocytes	Marked: metaplasia and/or fragmentation of one meniscus, osteophyte formation, synovitis/hyperplasia
3	2 + loss of articular cartilage <50% (e.g. erosion, flap, or callus)	Severe: metaplasia and/or fragmentation of both menisci, total loss of at least 1 articular surface (eburnation), osteophyte formation, advanced synovitis/hyperplasia
4	3 + fragmentation and fissuring in an area	N/A
5	4 + fragmentation and fissuring in >75% of the cartilage surface	N/A
6	Total loss of normal articular cartilage (end-stage)	N/A

Table S1. Safranin O scoring of tibial plateau cartilage degeneration and H&E scoring of overall stifle degenerative joint disease.

Water content into μPLs. To estimate the water content in the μPLs, a profilometric optical analysis was performed on samples before and after lyophilization. In both conditions, particle height and width were measured (**Table. S2**) (n = 8). The volume of the particles was calculated as Volume = Heigh × Width^2. Prior lyophylization the average μPL volume is 6680.30±198.40 μm^3, whereas post lyophilization the μPL volume is 6064.30±383.42 μm^3. This results in ~10%

difference in volume that should be ascribed to the water content. Notice that, given the small volume and mass of μPLs, a total weight analysis prior and post lyophilization using high precision laboratory scales would require several tens of billions of μPL, which is simply impractable at the current state of development. Also, notice that the profilometric analysis was performed under regular ambient conditions, thus the lyophilized samples may have gained already water from the air umidity. As such the 10% volume difference should be considered as a conservative, first approximation on the water content in the μPL. It is here also important to highlight that μPL are different from conventional solid, spherical PLGA microparticles that have a more compact structure. This was demonstrated in a previous work by the authors [2], where the 'compactness' of μPL was increased by increasing the total mass of PLGA used during the synthesis process. Indeed, a reduction in degradation rate and drug release rate were documented as the mass of PLGA was increased. This has to be ascribed to a progressive reduction in water content in the μPL with the increase in PLGA mass.

	Sample	Particles volume (μm3)
Pre Lyophilization	μPL 1	6570.38
	μPL 2	6715.10
	μPL 3	6757.17
	μPL 4	6987.39
	μPL 5	6432.72
	μPL 6	6567.36
	μPL 7	6498.02
	μPL 8	6914.28
Post Lyophilization	μPL 1	5853.12
	μPL 2	5688.47
	μPL 3	5553.17
	μPL 4	6050.67
	μPL 5	5890.31
	μPL 6	6483.02
	μPL 7	6485.71
	μPL 8	6509.93

Table S2. microplate volumes prior and after lyophilization via optical profilometric analysis.

SUPPORTING RESULTS

Statistically significant differences for all the experimental groups of **Figure 3a** were assessed and listed in the **Table S3**. Significance corresponds to a p-value smaller than 0.05.

	**p values**		
DEX Concentration (μM)	Free DEX *vs* μPLs	Free DEX *vs* DEX-μPLs	μPLs *vs* DEX-μPLs
0.01	0.0012	0.0027	0.2739
0.50	0.6650	0.2169	0.2958
1	0.0611	0.2874	0.1340
10	0.0288	0.0508	0.7174
30	0.0002	0.0019	0.9644

Table S4. List of p-values for all the experimental groups of **Figure 5**.

Statistically significant differences for all the experimental groups of **Figure 5c** were assessed and listed in the **Table S4**. Significance corresponds to a p-value smaller than 0.05.

Gene	Healthy (yellow triangle) vs free DEX (blue circle)	Healthy (yellow triangle) vs Empty µPL (green triangle)	Healthy (yellow triangle) vs DEX- µPL (red square)	Saline (black cross) vs free DEX (blue circle)	Saline (black cross) vs Empty µPL (green triangle)	Saline (black cross) vs DEX- µPL (red square)	Free DEX (blue circle) vs Empty µPL (green triangle)	Free DEX (blue circle) vs DEX- µPL (red square)	Empty µPL (green triangle) vs DEX- µPL (red square)
**IL-1β**	0.0007	0.0015	0.0096	0.5658	0.2760	0.0013	0.5754	0.0024	0.0070
**TNF-α**	0.000061	0.0129	0.0013	0.7827	0.05889	0.0222	0.0612	0.0244	0.9559
**IL-6**	0.0006	0.0004	0.0220	0.34401	0.1909	0.0083	0.7281	0.0376	0.0506
**MMP13**	0.00005	0.00007	0.0044	0.3982	0.0086	0.0053	0.0268	0.0107	0.2614

Table S3. List of p-values for all the experimental groups of **Figure 5**.

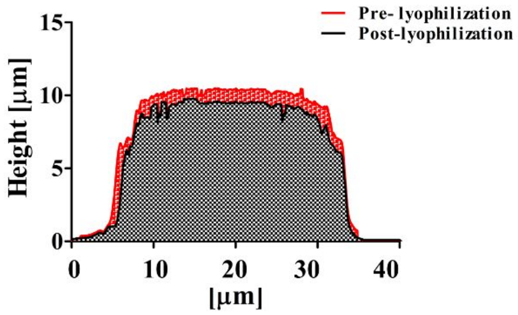

Figure S1. A profilometric analysis of hydrated and lyophilized µPLs. Representative cross-sectional profiles of µPLs prior (red contour) and after (black contour) lyophilization.

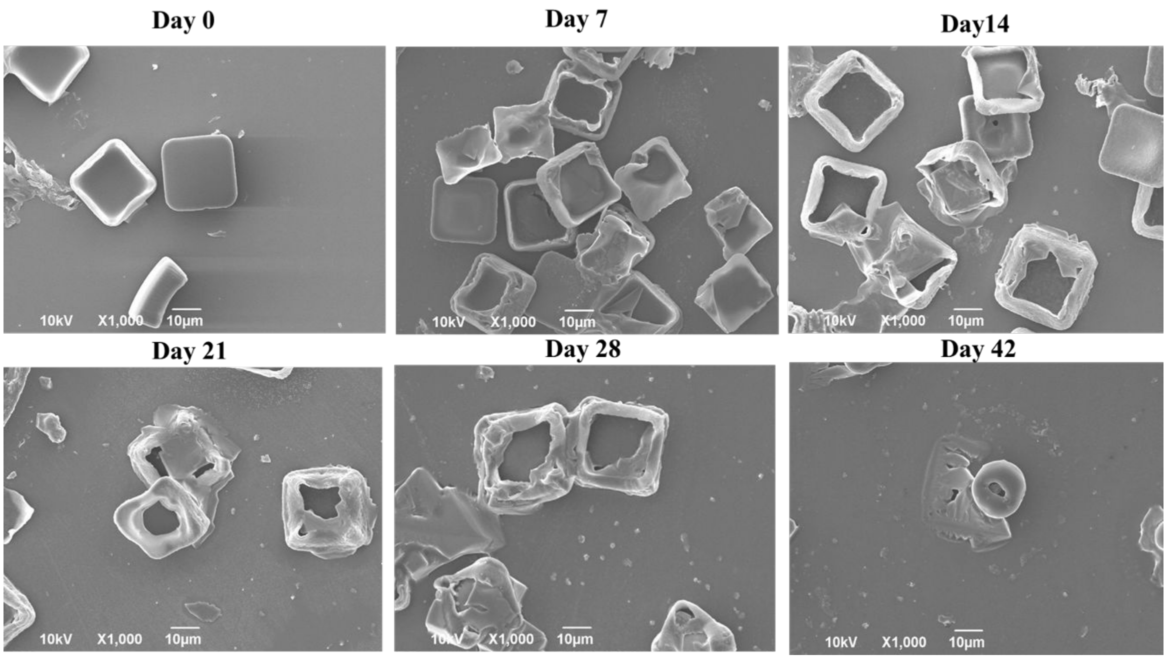

Figure S2. Particle degradation. µPLs degradation under physiological conditions, monitored over time using scanning electron microscopy.

a b

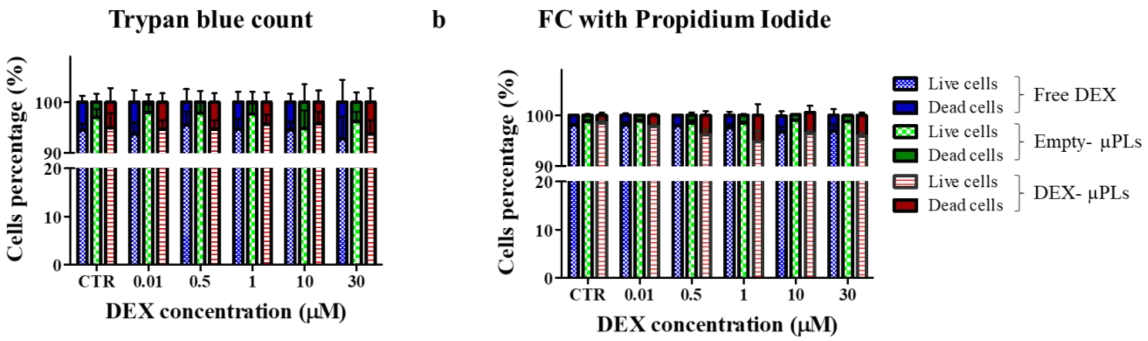

c

	Trypan blue			FC		
	Live cells			Live cells		
DEX Concentration (µM)	Free DEX	µPLs	DEX- µPLs	Free DEX	µPLs	DEX- µPLs
CTR	94.50 ± 1.29	97.00 ± 1.67	95.00 ± 2.83	98.13 ± 0.23	98.83 ± 0.28	98.59 ± 0.54
0.01	93.67 ± 2.42	98.00 ± 1.55	94.67 ± 1.75	98.14 ± 0.46	96.97 ± 0.23	97.90 ± 0.33
0.5	95.50 ± 2.59	97.80 ± 2.28	94.67 ± 1.75	97.92 ± 0.10	98.50 ± 0.62	96.23 ± 0.88
1	94.67 ± 2.07	97.67 ± 2.07	95.67 ± 1.97	97.51± 0.72	98.55 ± 0.27	94.79 ± 2.27
10	94.50 ± 1.64	94.83 ± 3.54	95.83 ± 2.32	96.72 ± 1.04	99.01 ± 0.12	96.53 ± 1.32
30	92.75 ± 4.42	96.17 ± 1.94	93.67 ± 2.80	96.94 ± 1.28	98.82 ± 0.13	95.94 ± 0.64
	Dead cells			Dead cells		
	Free DEX	µPLs	DEX- µPLs	Free DEX	µPLs	DEX- µPLs
CTR	5.50 ± 1.29	3.00 ± 1.67	5.00 ± 2.83	1.87 ± 0.23	1.17 ± 0.28	1.41 ± 0.54
0.01	6.33 ± 2.42	2.00 ± 1.55	5.33 ± 1.75	1.86 ± 0.46	1.03 ± 0.23	2.10 ± 0.33
0.5	4.50 ± 2.59	2.20 ± 2.28	5.33 ± 1.75	2.08 ± 0.10	1.50 ± 0.62	3.76 ± 0.88
1	5.33 ± 2.07	2.33 ± 2.07	4.33 ± 1.97	2.50 ± 0.72	1.45 ± 0.27	5.21 ± 2.27
10	5.50 ± 1.64	5.17 ± 3.54	4.17 ± 2.32	3.12 ± 1.04	1.10 ± 0.12	4.02 ± 1.32
30	7.25 ± 4.42	3.83 ± 1.94	6.33 ± 2.80	3.05 ± 1.28	1.18 ± 0.13	4.06 ± 0.64

Figure S3. Determination of live/dead cells after exposure to dexamethasone-loaded microPlates (DEX–µPLs). a. Percentage of live/dead cells after 24h exposure to different concentrations of free DEX, DEX–µPLs and empty µPLs using the trypan blue dye; **b.** Percentage of live/dead cells after 24h exposure to different concentrations of free DEX, DEX–µPLs and empty µPLs using FC with Propidium Iodide; **c.** Percentage of live/dead cells obtained with both techniques. Results are expressed as the average ± SD (n = 3).

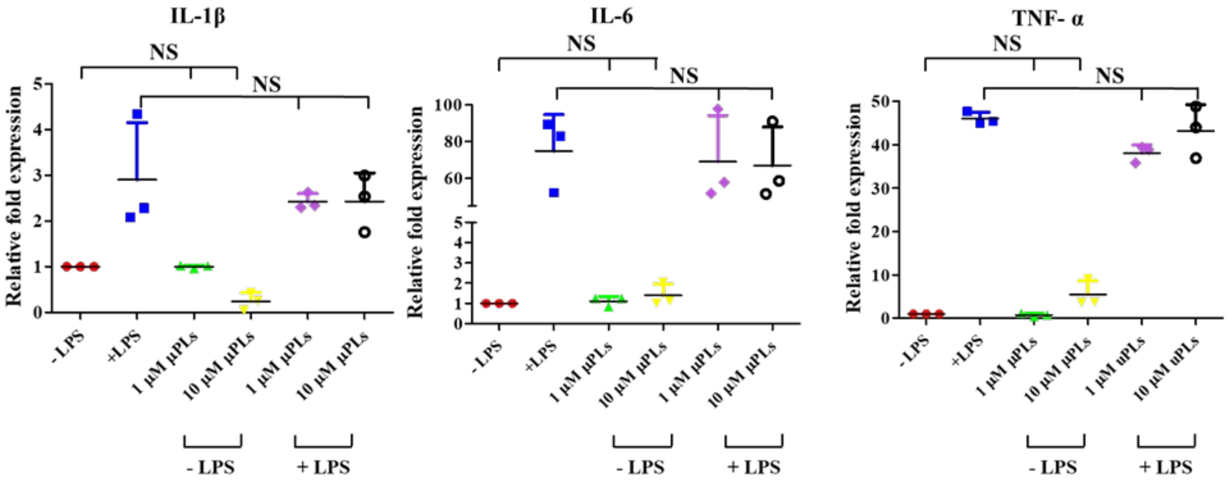

Figure S4: *In vitro* gene expression effect of empty microPlates (μPLs). Expression levels of pro-inflammatory cytokines IL-1β, IL-6, and TNF-α in ATDC5 cells with or without LPS stimulation. Results are presented as average ± SD (n = 3). Multiple comparisons were performed using, as post hoc test, the Tukey's significant difference (HSD) test.

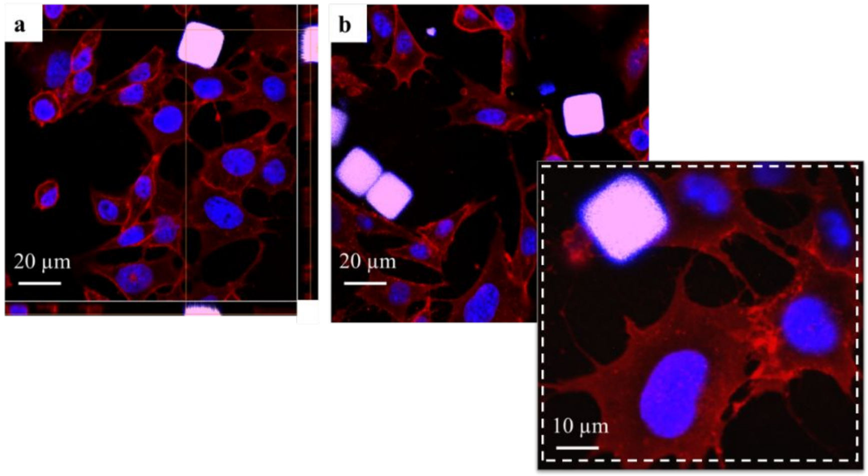

Figure S5. ATDC5 cells interaction with µPLs. a. 3D and **b.** confocal microscopy analysis of ATDC5 cells incubated with CURC - µPLs.

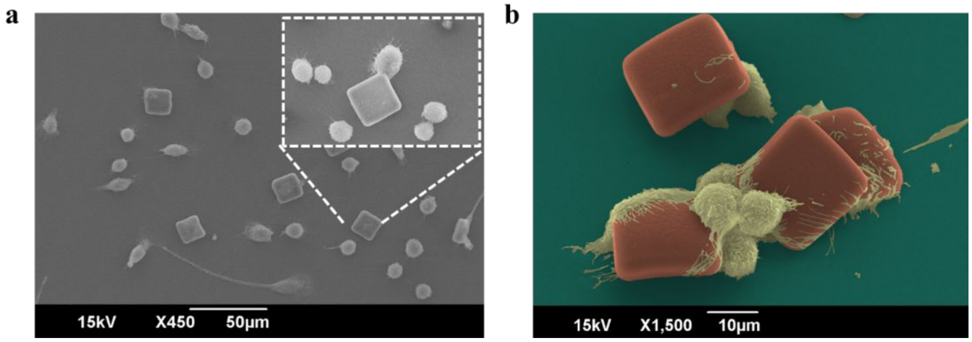

Figure S6. RAW 264.7 macrophages interacting with µPLs. a. SEM images of RAW 264.7 macrophages incubated with µPLs. The lateral inset shows a magnified image of cells surrouding the µPL without internalizing it; **b.** false-color SEM image of µPLs (red / orange) surroudned by RAW 264.7 macrophages (light beige).

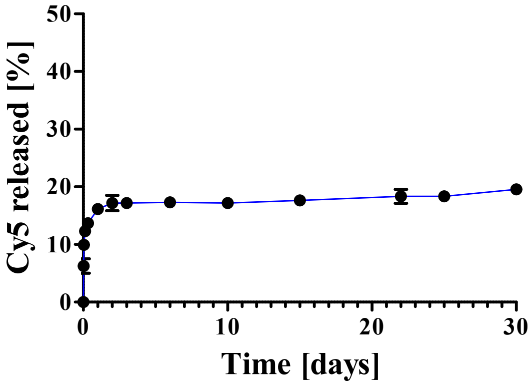

Figure S7. Cy5–μPL stability. The release of Cy5 molecules from Cy5–μPLs is plotted over the full period of observation (30 days) at 37 °C. The direct conjugation of Cy5 molecules to the PLGA structure of μPLs is extremely stable with a moderate loss less than 20% documented within the first hours only.

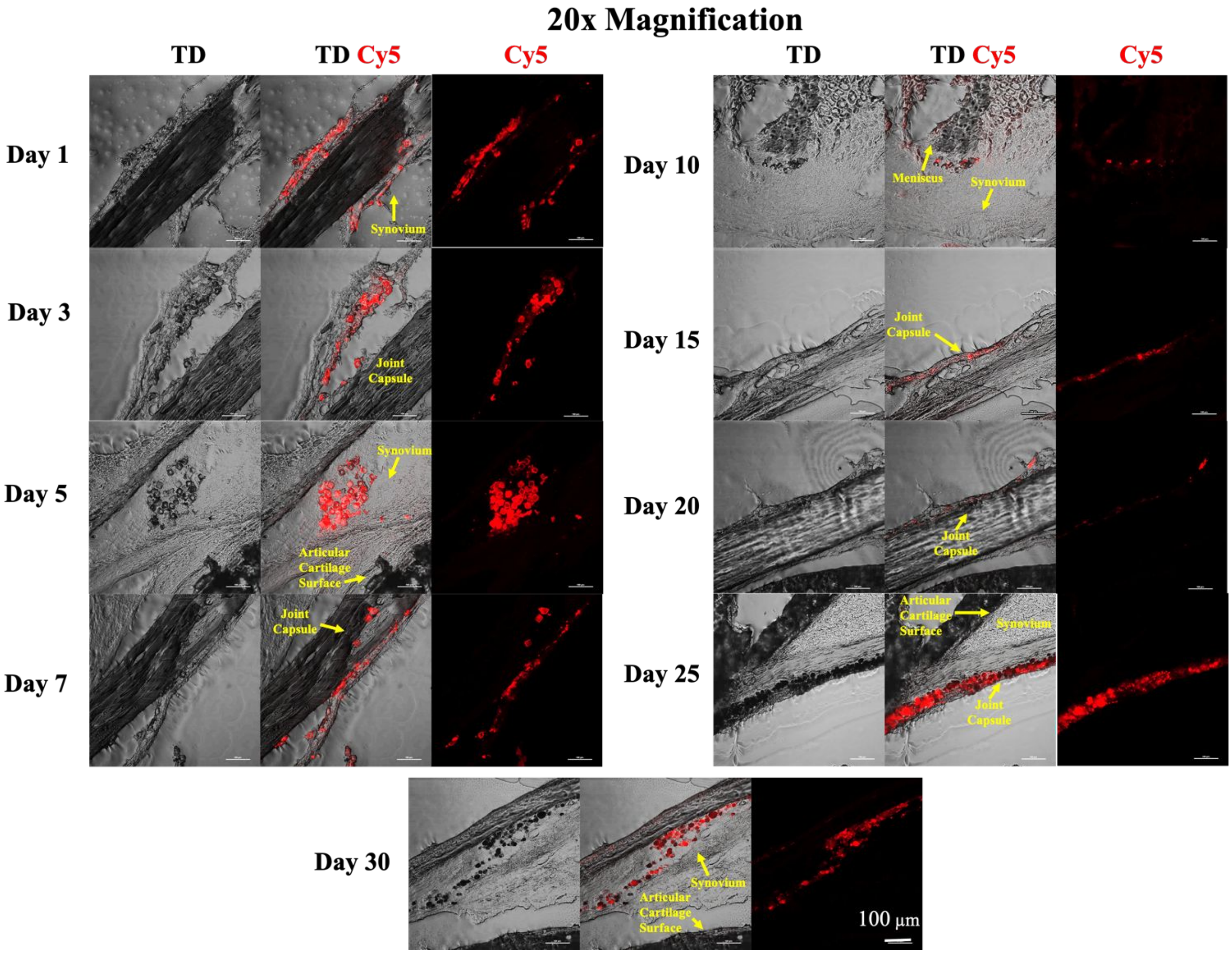

Figure S8. 20x Images of Cy5- μPLs within the knee joint of a mouse model of PTOA at multiple time points. Cy5-μPLs are seen in the joint for up to 30 days. Surface erosion and loss of shape and structure of microplates is evident over time. TD = Transmission Detector. In all images scale bar = 100 – μm.

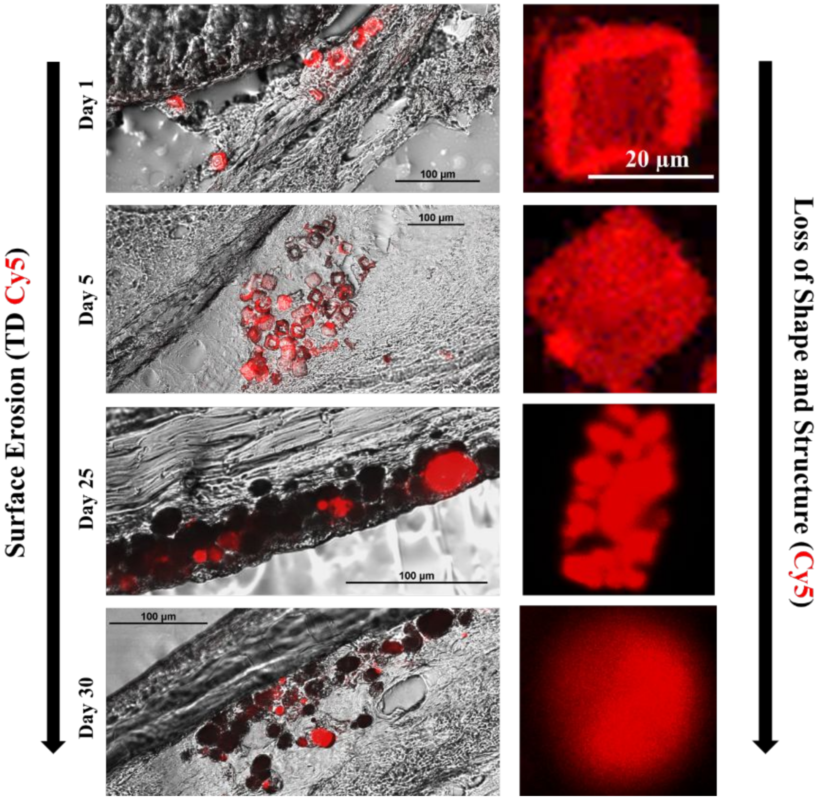

Figure S9. Magnified images of Cy5-µPLs within the knee joint of a mouse model of PTOA at multiple time points. 20x Images zoomed in on Cy5-µPLs within a mouse model of PTOA at days 1, 5, 25, and 30. Microplates are seen in the joint for up to 30 days with surface erosion evident by loss of fluorescence (heterogenous) in a significant number of individual Cy5-µPLs at days 25 and 30 as compared to days 1 and 5. Cy5-µPLs at days 1 and 5 have a consistent square shape and structure meanwhile Cy5-µPLs at days 25 and 30 have lost this morphology. In all images on the right scale bar = 100 µm. TD = Transmission Detector.

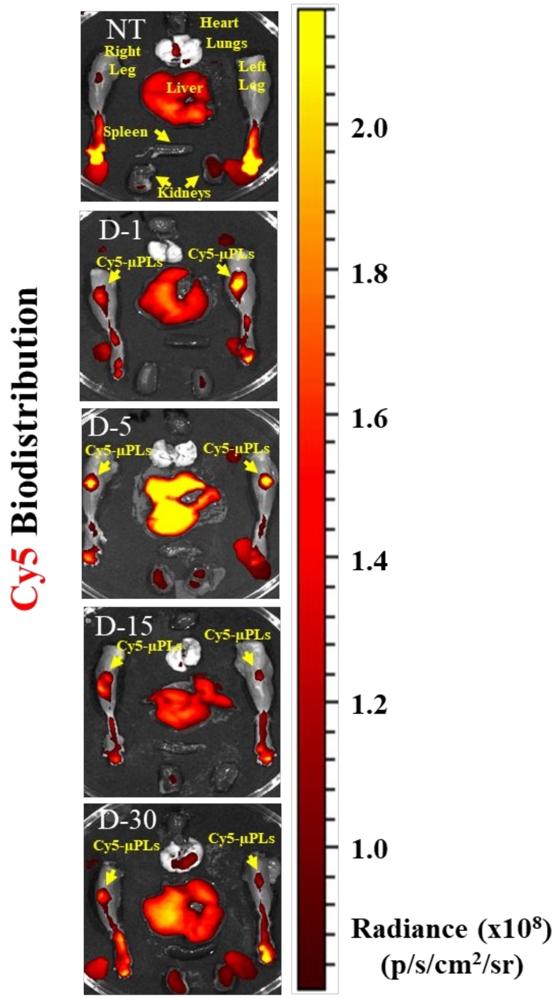

Figure S10. Cy5-µPLs *ex-vivo* organ biodistribution in a mouse model of PTOA at multiple time points. Note that the fluorescent signal in the liver, which is seen even in the no-treatment (NT) mice, has to be ascribed to the autofluorescence resulting from a regular mouse diet. Paw autofluorescence can be ascribed to skin being left on the paw. (D-#, where # represents days after intra-articular injection). (N = 1-2 mice, 2-4 limbs per time point).

Supporting References

(1) Aigner, T.; Söder, S. Histopathological Examination of Joint Degeneration: Typing, Grading and Staging of Osteoarthritis. Der Pathologe 2006, 27 (6), 431.

(2) Di Francesco, M.; Primavera, R.; Summa, M.; Pannuzzo, M.; Di Francesco, V.; Di Mascolo, D.; Bertorelli, R.; Decuzzi, P. Engineering Shape-Defined Plga Microplates for

the Sustained Release of Anti-Inflammatory Molecules. Journal of Controlled Release 2020, 319, 201-212.
